# Cytoplasmic and nuclear NFATc3 cooperatively contributes to vascular smooth muscle cell dysfunction and drives aortic aneurysm and dissection

**DOI:** 10.1016/j.apsb.2025.05.016

**Published:** 2025-05-21

**Authors:** Xiu Liu, Li Zhao, Deshen Liu, Lingna Zhao, Yonghua Tuo, Qinbao Peng, Fangze Huang, Zhengkun Song, Chuanjie Niu, Xiaoxia He, Yu Xu, Jun Wan, Peng Zhu, Zhengyang Jian, Jiawei Guo, Yingying Liu, Jun Lu, Sijia Liang, Shaoyi Zheng

**Affiliations:** a Department of Cardiovascular Surgery, Nanfang Hospital, Southern Medical University, Guangzhou 510515, China; b Department of Neurosurgery, the Second Affiliated Hospital of Guangzhou Medical University, Guangzhou 510260, China; c Center for Drug Inspection of Guizhou Medical Products Administration, Guiyang 550081, China; d Department of Pharmacology, School of Medicine, Yangtze University, Jingzhou 434023, China; e Guangzhou Women and Children’s Medical Center, Guangdong Provincial Clinical Research Center for Child Health, Guangzhou 510623, China; f Department of Pharmacology, Zhongshan School of Medicine, Sun Yat-sen University, Guangzhou 510080, China

**Keywords:** Aortic aneurysm and dissection, NFATc3, eEF2, Translational elongation, Extracellular matrix degradation, MMP2, MMP9

## Abstract

This study investigated the role of the nuclear factor of activated T cells c3 (NFATc3) in vascular smooth muscle cells (VSMCs) during aortic aneurysm and dissection (AAD) progression and the underlying molecular mechanisms. Cytoplasmic and nuclear NFATc3 levels were elevated in human and mouse AAD. VSMC–NFATc3 deletion reduced thoracic AAD (TAAD) and abdominal aortic aneurysm (AAA) progression in mice, contrary to VSMC–NFATc3 overexpression. VSMC–NFATc3 deletion reduced extracellular matrix (ECM) degradation and maintained the VSMC contractile phenotype. Nuclear NFATc3 targeted and transcriptionally upregulated matrix metalloproteinase 9 (MMP9) and MMP2, promoting ECM degradation and AAD development. NFATc3 promoted VSMC phenotypic switching by binding to eukaryotic elongation factor 2 (eEF2) and inhibiting its phosphorylation in the VSMC cytoplasm. Restoring eEF2 reversed the beneficial effects in VSMC-specific NFATc3-knockout mice. Cabamiquine—targets eEF2 and inhibits protein synthesis—inhibited AAD development and progression in VSMC-NFATc3-overexpressing mice. VSMC–NFATc3 promoted VSMC switch and ECM degradation while exacerbating AAD development, making it a novel potential therapeutic target for preventing and treating AAD.

## Graphical abstract

Nuclear NFATc3 upregulates MMP9/MMP2 to promote ECM degradation, while cytoplasmic NFATc3 inhibits eEF2 phosphorylation to drive VSMC phenotypic switching, collectively causing VSMC dysfunction and accelerating AAD progression.

**Figure:**
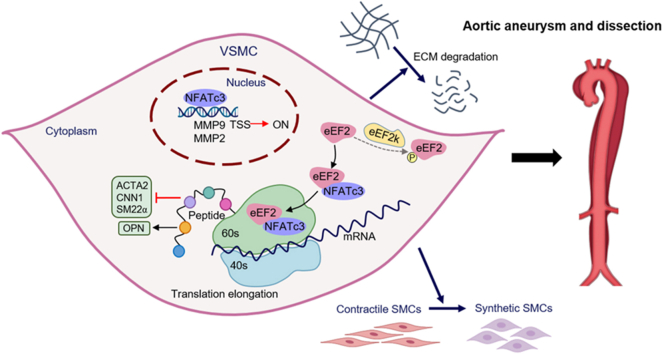

## 1 Introduction

Thoracic aortic aneurysm and dissection (TAAD) and abdominal aortic aneurysm (AAA) are fatal diseases resulting in catastrophic aortic rupture and sudden death^1^. TAAD and AAA exhibit several pathogenic similarities, including extracellular matrix (ECM) depletion, progressive vascular smooth muscle cell (VSMC) phenotypic switching, VSMC loss, and chronic inflammatory conditions^2^. Genetic disorders cause most TAAD cases, and TAAD-causing mutations have been identified in fibrillin-1, collagen type III alpha 1, transforming growth factor (TGF) B2, TGFB3, TGF-*β* receptor (TGFBR), *α*-smooth muscle actin (ACTA2), mothers against decapentaplegic homolog 3 (SMAD3), myosin heavy chain 11 (MYH11), and solute carrier family two member 10^3^. AAA risk factors include age, male sex, hypertension, smoking, elevated cholesterol levels, pregnancy, and atherosclerosis^4^. ECM degradation is primarily caused by increased matrix metalloproteinases (MMPs). Most patients with aortic aneurysm and dissection (AAD) are asymptomatic before fatal rupture, impeding early diagnosis^5^. Although effective drugs are available to prevent aortic degeneration or slow disease progression, the underlying pathological mechanism of AAD remains unknown. Moreover, VSMC phenotypic transformation contributes to structural aortic abnormalities, including degradation and degeneration, making VSMCs a potential therapeutic target.

The nuclear factor of activated T cells (NFAT) family of proteins was first identified *via* transcription factor activation by Ca^2+^/calcineurin signaling in T cells^6^. NFATs include four well-characterized members (NFATc1–c4) that are critical in regulating cytokine expression in T cells^6^. NFATs have various roles outside the immune system and are critical regulators in cardiovascular diseases, including vascular development and cardiac hypertrophy^7^, ^8^, ^9^. Additionally, NFATs are crucial for smooth muscle cell functions^10^^,^^11^. However, the role of individual NFAT isoforms in VSMC function remains unclear.

NFATc3 is a critical transcription factor belonging to the NFAT family that plays a pivotal role in calcium-dependent gene regulation. While initially studied for its role in T cell activation and immune response^12^, NFATc3 has since emerged to play a key role in cardiovascular physiology, particularly in heart diseases. Its activation is mediated by calcium signaling pathways, which trigger its dephosphorylation and translocation into the nucleus, where it regulates the expression of genes involved in cellular growth, survival, and differentiation^6^.

NFATc3 contributes to pathological cardiac remodeling, a process characterized by hypertrophy, fibrosis, and eventually heart failure. NFATc3 regulates cardiac hypertrophy by promoting the expression of hypertrophic genes in response to mechanical stress and neurohumoral stimuli, NFATc3 activation upregulates hypertrophic markers, thereby exacerbating myocardial thickening and leading to diminished cardiac function^13^^,^^14^. We previously found that macrophage NFATc3 inhibits atherosclerosis and foam cell formation^15^, however, the precise role of NFATc3 in AAD remains unknown. Here, we re-analyzed single-cell RNA sequencing data from the Gene Expression Omnibus (GEO) database, which included samples from patients with healthy ascending aortic tissue and those diagnosed with thoracic aortic aneurysm (TAA). Our results showed significant upregulation of NFATc3 in smooth muscle cells (SMCs) in the TAA group compared to healthy controls. Similarly, we observed NFATc3 upregulation in VSMCs from AAD tissues, suggesting a potential role for NFATc3 in aortic degeneration. Given these findings, in this study, we investigated whether NFATc3 is involved in aortic degeneration and AAD development using human samples as well as animal models. We analyzed 80 human aortic samples, including 36 control samples, 36 TAAD samples, and 8 abdominal aortic aneurysm samples. Additionally, we employed VSMC–NFATc3 conditional knockout and knock-in mice in different AAD models to further explore the role of NFATc3 in aortic degeneration. We found that cytoplasmic NFATc3 is critical for promoting the VSMC phenotypic switch by directly binding to eukaryotic elongation factor 2 (eEF2) and inhibiting its phosphorylation, while nuclear NFATc3 transcriptionally upregulates matrix metalloproteinase 2 (MMP2) and MMP9, thereby enhancing ECM degradation. These combined effects exacerbate AAD development. These findings collectively suggest that NFATc3 is a key regulator of VSMC phenotype switching and ECM degradation, representing a potential therapeutic target for preventing and treating AAD.

## 2 Materials and methods

### 2.1 Human samples

All human aortic samples (*n* = 80) were processed under protocols approved by the institutional review board at Nanfang Hospital of Southern Medical University. Human aortic dissection and aneurysm samples were obtained from the Nanfang Hospital of Southern Medical University, Guangzhou, China. All experiments involving the use of human samples were approved by the Ethical Committee of Nanfang Hospital (approval No. NFEC-2022-257), and all participants were recruited after obtaining informed consent. The medical information of all participants is detailed in Supporting Information Table S1.

Human TAAD samples (*n* = 36) were obtained from patients who met the TAAD diagnostic criteria according to the 2022 American College of Cardiology (ACC)/American Heart Association (AHA) Guidelines for the Diagnosis and Management of Aortic Diseases and underwent emergent aortic repair (Sun’s procedure) under cardiopulmonary bypass. The patients were excluded if they had a heritable form of aortopathy (*e*.*g*., Marfan syndrome, Loeys–Dietz syndrome, Ehlers–Danlos syndrome) or bicuspid aortic valve. Human AAA tissue samples (*n* = 8) were obtained from patients undergoing surgical repair for AAA, and all patients with an abdominal aortic diameter of ≥55 mm were recruited. The aortic status of all subjects was evaluated using tomography angiography. Control aortic tissue (*n* = 36) was collected from age-matched patients undergoing coronary bypass without aortic aneurysm, dissection, coarctation, or previous aortic repair. Samples acquired from the same site were compared. Hypertension was defined as a blood pressure of ≥140/90 mmHg or receiving treatment for hypertension before admission. Characteristics of patients and control individuals are shown in Table S1.

The aortic samples were shipped to our lab on wet ice and processed within 2 h of collection. The processing steps included removing periaortic fat and intraluminal thrombus from the aortic tissue, followed by rinsing with 0.9% normal saline. The aortic tissue was then divided into 4 portions. The first was fixed in 10% formalin and embedded in optimal cutting temperature compound (OCT) for histological analysis; the second was embedded in optimal cutting temperature compound for immunofluorescence staining; the third was placed in RNAlater for RNA analysis; and the fourth was snap-frozen in liquid nitrogen for protein extraction. All experiments were conducted in accordance with the principles of the Declaration of Helsinki.

### 2.2 Animal models

All animal experiments were performed according to the Animal Research policies of the Southern Medical University Committee in Nanfang Hospital (approval No. NFYY-2021-0731) and conformed to the Guide for the Care and Use of Laboratory Animals of the National Institute of Health in China.

NFATc3-fled (*Nfatc3*^fl/fl^) mice were generated at GemPharmatech Co., Ltd. (Jiangsu, China) using the CRISPR-Cas9 system by introducing loxP inserts flanking exons 2 and 3. Two gRNAs (gRNA1: 5′-GTCTTTACGAACAGTAAACC-3′; gRNA2: 5′-GTACTCGCCCTTTATGTATG-3′) were synthesized and cloned into a gRNA expression vector. The vector was mixed with Cas9 mRNA and single-stranded donor DNAs, followed by microinjection into zygotes derived from C57BL/6J mice. After confirming that the two lox*P* inserts were introduced into the intended sites on the founder mice *via* PCR, the offspring were bred with heterozygous *Nfatc3*^*fl+/−*^ mice to obtain homozygous *Nfatc3*^fl/fl^ mice.

The human *NFATC3* CDS conditional knock-in at the ROSA26 locus in C57BL/6J mice (*Nfatc3*-KI^fl/fl^) was performed *via* Cas9/CRISPR-mediated genome editing (Cyagen, Suzhou, China). For the NFATc3 knock-in model, the “CAG promoter-loxP-PGK-Neo-6∗SV40 pA-loxP-Kozak-Human NFATC3 CDS-rBG pA” cassette was cloned into intron 1 of *ROSA26*. To engineer the targeting vector, homology arms were generated *via* PCR using the BAC clone as template. Subsequently, Cas9 and gRNA were co-injected into fertilized eggs with the targeting vector for NFATc3 knock-in mouse production. Mouse genotypes were examined *via* PCR using total DNA derived from the tail tips. Genotyping PCR was performed to identify Rosa 5′LR using the primers listed in Supporting Information Table S2.

*Myh11*-Cre mice [B6.FVB-Tg (Myh11-icre/ERT2)1Soff/J; stock no. 019079] were purchased from Jackson Laboratory (Bar Harbor, ME, USA) and backcrossed to the C57BL/6J genetic background for nine generations. VSMC-specific NFATc3-knockout mice (*Nfatc3*^smcKO^) were generated by crossing *Nfatc3*^fl/fl^ and *Myh11*-Cre mice and treating them with tamoxifen to induce Cre expression. VSMC-specific *Nfatc3* knock-in mice (*Nfatc3*^smcKI^) were generated by crossing *Nfatc3*-KI^fl/fl^ mice with *Myh11*-Cre mice and treating them with tamoxifen to induce Cre expression. The subsequent generations of mice were genotyped using two pairs of specific primers and a pair of Cre-specific primers listed in Table S2.

#### 2.2.1 Tamoxifen injection

Tamoxifen (20 mg/mL; T5648, Sigma, St. Louis, MO, USA) was dissolved in corn oil (#C8267, Sigma) and administered intraperitoneally to 3- or 6-week-old mice at 75 mg/kg/day for 5 consecutive days. Different AAD mouse models were established 2 weeks after tamoxifen administration.

#### 2.2.2 Mouse β-aminopropionitrile (BAPN)/angiotensin II (AngII) model

Five-week-old male mice were given water containing BAPN (0.3 g/kg/day) (HY-Y1750, Med Chem Express, Monmouth Junction, NJ, USA) for 28 days, and then infused with AngII (1000 ng/kg/min) (HY-13948, Med Chem Express) for 3 days with a mini-pump (model 1003D, Alzet, Cupertino, CA, USA).

#### 2.2.3 Mouse Pcsk9^DY^/AngII model

Eight-week-old male mice were injected with adeno-associated virus (AAV)-LacZ or AAV-*Pcsk9*^DY^ (2 × 10^11^ genomic copies) and fed a Western-type diet for 6 weeks. Two weeks post-AAV injection, the mice were infused with AngII (1500 ng/kg/min) (HY-13948, Med Chem Express) for the last 4 weeks of the experiment with a mini-pump (model 2004, Alzet).

#### 2.2.4 Mouse BAPN model

Five-week-old male mice were given water containing BAPN (0.6 g/kg/day) (HY-Y1750, Med Chem Express) for 28 days.

In the 3 AAD mouse models, we measured the diameters of the ascending, arch, and abdominal segments of the aorta using DP2-BSW software (Olympus Life Science Solutions, Center Valley, PA, USA) in a double-blinded manner. Aneurysm was defined as aortic dilation >50% of the normal aorta. The mice died of aneurysm dissection or rupture, confirmed through post-mortem analysis showing aortic wall rupture, histological examination revealing disrupted vascular structure, and pre-mortem imaging detecting aneurysm progression followed by sudden death. Mice confirmed to have died from aortic rupture or dissection was not subjected to aortic diameter analysis, histological examinations, or serum lipid level analysis; instead, they were analyzed for AAD incidence. Sample sizes were determined on the basis of previous experimental experience and extensive review of the literature in the field. For experiments involving pharmacologic treatment to mice, mice of each genotype were randomized into control or treatment groups such that littermates were dispersed into separate groups whenever possible; the information of animal experiments is detailed in Supporting Information Table S3. The mice that died from aortic rupture were not included in the subsequent statistical analysis of aortic diameter as well as serum, elastin degradation, and collagen content analyses. Investigators responsible for analyzing samples were blinded to the identity of the genotype, treatment, and disease state of the individuals from which the samples were derived. “*n*” refers to biological replicates.

All mice were kept under a 12 h light/dark cycle at 23 °C. Male mice aged 8 weeks were fed a chow diet or high-fat diet (HFD, 40% kcal fat, 10.5% kcal sucrose, 1.25% cholesterol; XT108C; Jiangsu Xietong Pharmaceutical Bio-engineering, Nanjing, China) for 8 weeks.

### 2.3 scRNA-seq analysis

To examine the distribution of NFATc3 expression in different types of vascular cells, 11 single-cell transcriptome datasets [3 control and 8 ascending thoracic aortic aneurysm (TAA) samples] from the GSE155468 dataset (48,128 individual cells) generated from human ascending aortic wall tissue were downloaded from the GEO database (GSE131778) and processed with the R package “Seurat” (v.4.3.0.1) for quality control, dimensionality reduction, and clustering. Genes expressed in fewer than 5 cells, as well as cells expressing fewer than 200 or more than 5000 genes or containing >10% mitochondrial genes were removed. *t*-SNE (distributed stochastic neighbor embedding) plots were generated for visualization of different cell clusters. Using the single-cell trajectory analysis method, 8 major cell types were annotated based on classical cell markers. Normalized data were used to visualize NFATc3 expression in different cell types. The random sampling approach was employed to select 100 cells from both the control (Con) and TAA groups to make the results more robust. Student’s *t*-test was used to analyze statistical differences between 2 groups, and statistical significance was set at *P* < 0.05.

### 2.4 RNA sequencing

Using the TRIzol reagent (Invitrogen, Carlsbad, CA, USA), total RNA was extracted from 9-week-old *Nfatc3*^fl/fl^ and *Nfatc3*^smcKO^ mice administered BAPN *via* drinking water for 4 weeks. RNA integrity was evaluated using the RNA Nano 6000 assay kit of the Agilent Bioanalyzer 2100 system (Agilent Technologies, Santa Clara, CA, USA). Library construction and sequencing were performed by Novogene (Beijing, China). RNA sequencing was performed following the manufacturer’s instructions, and 1 μg of RNA was utilized as input material for library preparation using the NEBNext Ultra RNA library prep kit for Illumina (New England Biolabs, Ipswich, MA, USA). The library quality was assessed using the Agilent Bioanalyzer 2100 system. DNA fragments were aligned using 150-bp paired-end sequencing on an Illumina HiSeq 2500 platform. Differential expression analysis was conducted using the DESeq2 R package. Genes with an adjusted *P*-value <0.05 and |fold-change| ≥ 2 were considered differentially expressed genes. Multiple test correction was performed using the Benjamini–Hochberg method. Gene Ontology enrichment analysis of differentially expressed genes was conducted using the clusterProfiler R package, which corrects gene length bias.

### 2.5 Mass spectrometry

MASMCs lysates were subjected to overnight immunoprecipitation using NFATc3 antibody (#4389) and control rabbit IgG antibody (#2729) (Cell Signaling Technology, Danvers, MS, USA), followed by conjugation with protein A/G-plus agarose beads for an additional 4 h. Gel electrophoresis was performed to prepare samples for subsequent proteomic analysis. The gels were silver-stained using a polyacrylamide gel electrophoresis (PAGE) gel silver staining kit (Solarbio, Beijing, China). To identify proteins interacting with NFATc3, LC–MS/MS analysis was conducted by Novogene (Beijing, China). Briefly, the co-immunoprecipitated gel bands and their corresponding negative gel bands were excised and digested with in-gel trypsin. The resulting peptide mixtures were analyzed using an EASY-nLC 1200 UHPLC system (Thermo Fisher, Waltham, MA, USA) coupled with a Q exactive HF-X mass spectrometer (Thermo Fisher) equipped with a nanospray flex electrospray ionization source (Thermo Fisher). The spectrum of each peptide was matched against the UNIPROT human protein database using proteome discoverer 2.2 (PD 2.2, Thermo Fisher). The search parameters included a mass tolerance of 10 ppm for precursor ions, a fragment ion mass tolerance of 0.02 Da, carbamidomethylation as a fixed modification, and methionine (M) oxidation and N-terminal acetylation as variable modifications. Up to 2 missed cleavage sites were allowed. Proteins with at least 1 unique peptide and a false discovery rate of no more than 1.0% were selected. Gene ontology enrichment analysis of the identified proteins was performed using the Interproscan software.

### 2.6 Vasorelaxation assay

Vasorelaxation was assayed using an organ chamber (DMT 620M, Winnipeg, CA, USA) according to the manufacturer’s protocol. Briefly, following euthanasia *via* intraperitoneal injection of pentobarbital sodium, the thoracic aorta was carefully dissected, and the adventitia and excess fat were cleaned. Arterial segments (3–4 mm long) were mounted as a ring and stretched to a resting tension of 5 mN in an organ bath containing modified Kreb’s solution (mmol/L: NaCl: 137, KCl: 5.4, CaCl_2_: 2.0, MgCl_2_: 1.1, NaH_2_PO_4_: 0.4, glucose: 5.6, NaHCO_3_: 11.9; heparin: 10 U/mL; pH = 7.2) gassed with 95% O_2_ and 5% CO_2_ at 37 °C. After a 1-h equilibration period, segments were contracted using 60 mmol/L KCl to test the arterial integrity. Aortic segments were stimulated with increasing concentrations of phenylephrine (Phe, 1 × 10^−9^ to 1 × 10^−5^ mol/L), concentration–contraction curves were constructed, and the maximal Phe contraction was measured. The individual Phe concentration–response curves were further analyzed using a non-linear regression curve (best-fit sigmoidal dose–response curve, Sigmaplot). Endothelium-dependent relaxation was determined based on the ability of acetylcholine (1 × 10^−9^ to 1 × 10^−5^ mol/L) to relax arteries pre-contracted with Phe (10^−5^ mol/L). All vasoconstriction responses were expressed as a percentage of the maximal contraction induced by KCl (100 mol/L). Individual Ach relaxation curves were further analyzed using a non-linear regression best-fit sigmoidal dose–response curve.

### 2.7 Cell culture and transfection

Human aortic smooth muscle cells (HASMCs), Cos7 cells, and HEK293T cells were acquired from Cell Bank of the Chinese Academy of Sciences (Shanghai, China). HASMCs and HEK293T cells were cultured in high-glucose Dulbecco’s modified Eagle’s medium (DMEM; Gibco BRL, Grand Island, NY, USA) supplemented with 10% fetal bovine serum (FBS) (VivaCell, Shanghai, China), 100 U/mL penicillin, and 100 μg/mL streptomycin. Mouse aortic smooth muscle cells (MASMCs) were separated from 10-week-old wild-type, *Nfatc3*^fl/fl^, *Nfatc3*^smcKO^, *Nfatc3*-KI^fl/fl^, and *Nfatc3*^smcKI^ mice *via* collagenase digestion. VSMC purity was detected using ACTA2 immunofluorescence staining. Isolated VSMCs were preserved in low-glucose DMEM containing 10% FBS; unless otherwise specified, cells from generations 4–8 were used for further experiments. All cells were preserved at 37 °C in a humidified 5% CO_2_ incubator. The siRNA duplex against human *NFATc3* (Qiagen, Hilden, Germany; sense: 5′-GCCUUCGUCUCAGUUACAATT-3′, antisense: 5′-UUGUAACUGAGACGAAGGCTT-3′) was transiently transfected using the Hiperfect Transfection Reagent (Qiagen) according to the manufacturer’s instructions. A scramble siRNA (Qiagen) was used as a negative control.

### 2.8 Histological analyses

After euthanasia, mice were perfused with saline through the left ventricle. The aorta was dissected, fixed in 4% paraformaldehyde, embedded in OCT, and sectioned. OCT-embedded aortic slices were stained with hematoxylin and eosin, Masson, and Verhoeff–Van Gieson stains according to the manufacturer’s instructions.

For hematoxylin and eosin staining, sufficient hematoxylin was applied to completely cover the tissue section and incubated for 5 min. To remove excess stain, the slices were rinsed with distilled water twice. Later, enough bluing reagent was applied to completely cover the tissue section and incubated for 10–15 s. The slices were rinsed with distilled water twice. The slices were immersed in absolute alcohol, and the excess portion was wiped off. Enough eosin Y solution was applied to totally cover the tissue section and incubated for 2–3 min. Thereafter, the slices were rinsed with enough alcohol. The slices were dehydrated with absolute alcohol thrice. The sections were cleaned and sealed in synthetic resin.

For elastic fiber staining, the slices were placed in the working elastic staining solution for 15 min and rinsed using distilled water until there was no excess stain on the slices. The slices were soaked 15–20 times in the differentiating solution and rinsed using distilled water. The slices were observed under a microscope for proper differentiation. They were rinsed using distilled water, placed in sodium thiosulfate solution for 1 min, and rinsed in distilled water again. The slices were stained for 2–5 min using Van Gieson’s solution. The slices were rinsed with 95% alcohol twice and dehydrated in absolute alcohol. The sections were cleaned and sealed in synthetic resin. The degree of elastic fiber fragmentation was scored on a scale of 1–4 (1 = none, 2 = minimal, 3 = moderate, 4 = severe and rupture).

For Masson trichrome staining, the slices were stained with Weigert’s iron hematoxylin, followed by Ponceau acid fuchsin. After treatment with phosphomolybdic-phosphotungstic acid, the slices were stained with light green in acetic acid. The quantity of blue-green pixels as a percentage of the total pixels was used to determine the percentage of collagen. All images were acquired with an Olympus BX63 microscope digital camera (Olympus, Tokyo, Japan).

### 2.9 Immunofluorescence staining and imaging

For immunofluorescence staining, formaldehyde-fixed cells on dishes and OCT-embedded aortic sections on slides were permeabilized with 0.3% Triton X-100. Blocking with 5% (bovine serum albumin) BSA reduced non-specific staining. Slices and dishes were incubated with primary antibodies overnight at 4 °C, washed with phosphate-buffered saline (PBS), and incubated with secondary antibodies. Nuclei were stained with 4′,6-diamino-2-phenylindole. The antibodies used were as follows: anti-ACTA2 (1:100 dilution; 67735-1-Ig), anti-NFATc3 (1:100 dilution; 18222-1-AP), anti-fibronectin (1:50 dilution; 15613-1-AP), anti-collagen type I (COL1; 1:50 dilution; 14695-1-AP), anti-eukaryotic elongation factor 2 (eEF2; 1:50 dilution; 20107-1-AP), anti-IL-1 beta (1:100 dilution; 26048-1-AP), anti-TNF alpha (1:100 dilution; 60291-1-Ig) (Proteintech, Rosemont, IL, USA), anti-p-eEF2 (1:50 dilution; ab53114; Abcam, Cambridge, England), and anti-IL-6 (1:100 dilution; ab290735; Abcam). Slices and dishes were detected with a fluorescence confocal microscope (LSM 980, Carl Zeiss, Oberkochen, Germany).

### 2.10 qRT-PCR analysis

Total RNA was isolated from aortic tissue and VSMCs using TRIzol reagent (Invitrogen) and homogenized using the Lu ka sample freezing grinder LUKYM-I (LUKYM, Guangzhou, China). The quantity and quality of total RNA were determined using a NanoDrop ND-2000 spectrophotometer (NanoDrop, Wilmington, DE, USA). Subsequently, 2 μg of RNA was reverse-transcribed using the Evo M-MLV RT Premix for qPCR (AG11706, Accurate Biotechnology, Changsha, China), and real-time PCR was performed using the SYBR Green Premix Pro Taq HS qPCR Kit (AG11701, Accurate Biotechnology) on a MyiQ single colour real-time PCR detection system (Bio-Rad Laboratories, Hercules, CA, USA). The primer sequences used (Invitrogen) are shown in Table S2. The fold-change in relative gene expression was calculated using the 2^−ΔΔCT^ method, using *β*-actin as an internal control.

### 2.11 Western blot and immunoprecipitation

Protein extracts were prepared from isolated cells using radioimmunoprecipitation assay lysis buffer containing a protease inhibitor cocktail (Merck, Darmstadt, Germany) and quantified using a BCA assay kit (Thermo Fisher). Nuclear and cytoplasmic proteins were isolated using the PARIS kit (AM1921) according to the manufacturer’s instructions (Ambion, Carlsbad, CA, USA). Lysates containing equal amounts of total protein were subjected to 6%–12% sodium dodecyl sulfate–PAGE (SDS–PAGE) and transferred onto polyvinylidene fluoride membranes (Millipore, Burlington, MA, USA). The membranes were blocked using a blocking reagent [Tris-buffered saline with 5% (*w*/*v*) non-fat dry milk and 0.1% (*v*/*v*) Tween 20, pH 7.4] for 1 h at room temperature and then incubated with the following primary antibodies at 4 °C overnight: anti-NFATc3 (#4998; 1:1000), anti-lamin B1 (#13435; 1:1000), anti-phospho-eEF2 (Thr56) (#2331; 1:1000), anti-eEF2 (#2332; 1:1000), eEF2k (#3692; 1:1000) (Cell Signaling Technology), anti-LDL-R (sc-11824; 1:500; Santa Cruz Biotechnology, Dallas, TX, USA), anti-MMP2 (ab97779; 1:500), anti-MMP9 (ab76003; 1:1000), anti-RPS6 (ab225676; 1:1000), anti-RPL28 (ab254927; 1:500), anti-protein phosphatase 2A (PP2A; ab32065; 1:500), anti-Flag (ab205606; 1:500), anti-his (ab213204; 1:5000) (Abcam), anti-fibronectin (15613-1-AP; 1:2000), anti-COL1A2 (14695-1-AP; 1:1000), anti-ACTA2 (67735-1-Ig; 1:1000), anti-CNN1 (24855-1-AP; 1:1000), anti-SM22*α* (10493-1-AP; 1:1000), anti-OPN (22952-1-AP; 1:1000), anti-*α*-tubulin (66031-1-Ig; 1:2000), *β*-actin (66009-1-Ig; 1:2000), GAPDH (60004-1-Ig; 1:2000) (ProteinTech), and anti-puromycin (EQ0001; 1:1000) (Kerafast, Boston, MA, USA).

After incubation with the secondary HRP-linked anti-mouse IgG (#7076; 1:1000) or HRP-linked anti-rabbit IgG (#7074; 1:1000) (Cell Signaling Technology) antibodies for 1 h at room temperature, the blots were visualized using an ECL kit (Thermo Fisher) and quantified using ImageJ software (NIH, Bethesda, Rockville, MD, USA).

For the immunoprecipitation assay, cell lysates were adjusted to equal amounts of total protein (500 μg) and incubated with the NFATc3 antibody (#4389), control rabbit IgG antibody (#2729), eEF2 antibody (#2332) (Cell Signaling Technology), Flag antibody (Abcam, ab205606), and His antibody (Abcam, ab213204) overnight at 4 °C, followed by immunoprecipitation using Protein PLUS A/G Agarose (sc-2003; Santa Cruz Biotechnology). After several washes with ice-cold PBS, the immunoprecipitates were subjected to Western blot as described above.

### 2.12 Plasmid construction and transfection

The cDNA fragment encoding mouse full-length NFATc3 and truncated NFATc3 (1–415, 416–597, and 598–1076 aa) and mutant-NFATc3 (deletion of 416–597 aa) was subcloned into the pcDNA3.1^+^ plasmid for co-immunoprecipitation analysis. The full-length mouse NFATc3 coding sequence and fragments encoding functional domains (NHR, RHR, and C-terminal domains) were subcloned into a C-terminal Flag tag vector for expression in HEK293T cells. The full-length mouse eEF2-encoding sequence was subcloned into a C-terminal 6 × His pcDNA3.1^+^ vector for expression in HEK293T cells. The eEF2 T56D and T56A mutants were generated using site-directed mutagenesis. All primers used for plasmid construction are listed in Table S2. Plasmid transfection was performed using Lipofectamine 2000 reagent (Invitrogen) according to manufacturer instructions.

### 2.13 AAV and lentivirus construction and injection

The pAAV/D374Y-*hPcsk9* (*Pcsk9*^DY^) plasmid driven by the ApoEHCR-hAAT promoter was a gift from Dr. Bentzon (plasmid #58379, Addgene, Watertown, MA, USA). The plasmids were separately co-transfected with the pAAV2/8 *trans*-plasmid carrying the AAV *rep* and *cap* genes and the pAAV helper plasmid into HEK293T cells to generate the AAV8 adenoviruses (AAV-*Pcsk9*^DY^). AAV-LacZ was used as a negative control. Viral titers were measured *via* PCR using vector-specific primers. AAV-*Pcsk9*^DY^ was delivered *via* a single tail-vein injection at a dose of 2 × 10^11^ vector genomes/mouse. Starting from Day 1 after injection, the mice were fed a chow diet or HFD for 6 weeks.

The pLV3-CMV-eEF2 constructs and viral packaging plasmids (pSPAX2 and pMD2.G) were co-transfected into 293T cells using Lipofectamine 2000 (Invitrogen) following the manufacturer’s recommendations. After 12 h, the culture medium was replaced with a fresh medium. The viral particles were harvested after 48 h. Subsequently, lentivirus particles were used to infect the target cells. Protein and RNA expression was assessed through immunoblotting to validate the successful eEF2 overexpression.

For injection, four-week-old *Nfatc3*^smcKO^ mice were intravenously injected with 1.0 × 10^7^ transduction units of eEF2-expressing lentivirus or negative control lentivirus. Seven days after transfection, 5-week-old male mice were administered water containing BAPN (0.3 g/kg/day) for 28 days.

### 2.14 Ultrasound

Ultrasonography was performed on C57BL/6J male mice using a Vevo 2100 ultrasound system with a MS550 (40 MHz) transducer. Mice were anesthetized with isoflurane and placed in a supine position on a heated platform (37 °C). For thoracic aorta imaging, the stage was slightly tilted clockwise. The transducer was placed on the right edge of the sternum at a 60° angle relative to the anterior midline. For abdominal aorta imaging, the stage was flat, and the transducer was placed longitudinally, just below the sternum and xiphoid process. The transducer probe and the *X*- and *Y*-axis stage knob were adjusted to show the aorta clearly. Thereafter, one cine loop was stored. Finally, the analysis software (Vevo 2100) was started, and the ultrasound data were opened for statistical analysis.

### 2.15 Blood pressure

Baseline blood pressure was measured without anesthesia using a tail-cuff system (KEWBASIS, Nanjing, China). Each mouse was measured at least 3 times consecutively, and the mean value was calculated.

### 2.16 MMP activity

MMP activity in the aortic wall was measured using gelatin colocalized with quenched fluorescein (DQ gelatin; Invitrogen) as a substrate. Following the manufacturer’s protocol, *in situ* and *in vitro* zymography were performed using freshly cut frozen aortic sections (10 μm). The sections were incubated with a fluorogenic gelatin substrate (DQ gelatin, Molecular Probes) dissolved in a zymography buffer (25 mg/mL) containing 50 mmol/L Tris–HCl pH 7.4 and 15 mmol/L CaCl_2_. Gelatinase activity was detected as green fluorescence (530 nm) using confocal microscopy (LSM980, Carl Zeiss, Germany).

To evaluate the role of NFATc3 in BAPN-induced MMP activation, the ascending aortas of *Nfatc3*^fl/fl^ and *Nfatc3*^smcKO^ mice were isolated vehicle or BAPN treatment. The MMP activity in aortic tissue was detected *via* gel zymography. Protein samples (15 μg per lane) were loaded onto SDS–PAGE and then packed into Novex 10% zymogram plus (gelatin) protein gels for separation. Gels were electrophoresed at 100 V at 4 °C for 2 h. After electrophoresis, the gels were renatured in zymogram renaturing buffer (Thermo Fisher) for 30 min at room temperature and then incubated with zymogram developing buffer (Thermo Fisher) at 37 °C overnight. The gels were stained with Coomassie blue staining solution (0.05% Coomassie R250 in 30% ethanol, 10% acetic acid) for 3 h. Thereafter, the gels were destained 3 times for 30 min in a destaining solution (20% ethanol and 7.5% acetic acid). The presence of a clearly white band on a blue background indicated MMP activity. We used a chemistry analyzer (Chemray800, Rayto Life and Analytical Sciences Co., Ltd., Shenzhen, China) to obtain images of the stained gels.

### 2.17 Serum analysis

After an overnight fast, blood samples were obtained from anesthetized mice (pentobarbital sodium, i.p.) before sacrifice. Total serum cholesterol, triglyceride, low-density lipoprotein, and high-density lipoprotein levels were measured using a fully automated clinical chemistry analyzer (Chemray800, Rayto Life and Analytical Sciences Co., Ltd.).

### 2.18 Luciferase reporter assay

DNA fragments of the *Mmp9* and *Mmp2* promoter regions were amplified using PCR and then cloned into the pGL4.10 basic promoter vector (Promega, Madison, WI, USA). Mutations were introduced using a quickchange lightning site-directed mutagenesis kit (Agilent Technologies). Constructs with the correct sequence were transfected into HEK293T cells. After 48 h, luciferase activity was measured using the dual-luciferase reporter assay system (Promega). Firefly luciferase activity was normalized to the *Renilla* luciferase activity.

### 2.19 ChIP-qPCR

The ChIP assay was performed using the upstate biotechnology chip assay kit (Millipore). Briefly, MASMCs (1 × 10^7^) were cross-linked with 1% formaldehyde for 10 min at 37 °C. The reaction was stopped by adding 125 mmol/L of glycine for 5 min. Nuclei were isolated using lysis buffer and subsequently sonicated in a Bioruptor ultrasonicator (UCD-300; Diagenode, Denville, NJ, USA) for 30 min (30 s on/off) to generate chromatin samples with 200–500 bp fragments. Each sample was then subjected to immunoprecipitation overnight using the following antibodies: NFATc3 (18222-1-AP; 4 μg; ProteinTech), anti-H3K4me3 (ab8580; 1 μg), anti-H3K27ac (ab4729; 1 μg; Abcam), and control rabbit IgG (#2729; 4 μg; Cell Signaling Technology). Protein A Dynabeads (Invitrogen) were then added to the sonicated chromatin. Following the immunoprecipitation and immobilization of the immunocomplexes, the samples were incubated at 65 °C for 4 h to reverse the formaldehyde cross-linking. After a standard wash, the associated DNA was eluted and purified. QPCR was performed using specific primers (Table S2) surrounding the indicated promoter regions. No-antibody (input) and control rabbit IgG were used as controls. The qPCR data were normalized to the chromatin input and expressed as a percentage of the input.

### 2.20 RNA-binding protein immunoprecipitation (RIP)

The MASMCs were lysed using RIP lysis buffer (Millipore). The supernatant was incubated with anti-NFATc3 antibody (18222-1-AP; 4 μg; ProteinTech) or control rabbit IgG antibody (#2729; 4 μg; Cell Signaling Technology) at 4 °C overnight. A/G magnetic beads were added to the supernatants, which were then incubated for another 6 h. After immobilizing the magnetic beads to the immune complexes with a magnetic separator (Millipore), RNA was extracted from the immunoprecipitates. cDNA was synthesized *via* reverse transcription and subjected to RT-PCR for target gene amplification.

### 2.21 Nuclear run-on assay

HASMCs were harvested, washed once with ice-cold PBS, lysed with the cold cell-lysis buffer [10 mmol/L Tris HCl (pH 7.4), 10 mmol/L NaCl, 3 mmol/L MgCl_2_, 0.5% (*v*/*v*) Nonidet P-40, and 0.5% (*v*/*v*) Triton-100], and centrifuged at 3000 × *g* for 10 min at 4 °C. After decanting the supernatant, the reaction buffer [10 mmol/L Tris HCl, (pH 8.0), 10 mmol/L NaCl, 5 mmol/L MgCl_2_, 50 mmol/L KCl, and 2.5 mmol/L NTP plus Biotin-16-UTP mix (Roche)] was added to the precipitated nuclei, and the samples were incubated at 30 °C for 30 min. Each sample was incubated with DNase I (200 U) for 20 min at 37 °C; then, the reaction was stopped by adding stop buffer (20 mg/mL proteinase K premixed with 10% sodium dodecyl sulfate at a ratio of 3:1), followed by further incubation at 37 °C for 15 min. Biotinylated nascent RNA was isolated using streptavidin beads (Active Motif, Carlsbad, CA, USA) and eluted in 10 mmol/L EDTA (pH 8.2) *via* incubation at 90 °C for 10 min. The eluted RNA was subjected to PCR analysis.

### 2.22 Mammalian two-hybrid assay

Fragments containing the functional domains (NHR, RHR, and C-terminal domains) of mouse NFATc3 were amplified *via* PCR and cloned into the pBIND vector using a one-step cloning kit (Vazyme, Nanjing, China). The cDNA inserts encoding mouse *eEF2* were subcloned into the pACT vector using the ClonExpress II one step cloning kit (Vazyme). HEK293T cells were co-transfected with the target and bait constructs along with the luciferase reporter plasmid pG5-luc in a 1:1:1 ratio. The cells were harvested 48 h after transfection, and cell lysates were used for luciferase activity assays using the dual-luciferase reporter assay system (Promega). The primers used for sub-cloning are shown in Table S2.

### 2.23 Polyribosome profiling

The following steps were followed to prepare ribosome fractions from MASMCs. Cell pellets or tissues were lysed in ice-cold PMS buffer composed of 20 mmol/L Tris pH 7.5, 100 mmol/L KCl, 5 mmol/L MgCl_2_, 0.3% (*v*/*v*) NP-40, 100 U/mL of SUPERase-In RNase inhibitor (Invitrogen), 1% *w*/*v* protease inhibitor, 1 mmol/L PMSF, and 0.5 mmol/L DTT. The lysates were then centrifuged at 10,000 × *g* for 10 min at 4 °C. The resulting supernatants, referred to as post-mitochondrial supernatants, were collected as cytosolic cell extracts. Post-mitochondrial supernatants were layered onto 11 mL of a sucrose gradient ranging from 10% to 50% (*w*/*v*). The sucrose gradients were subjected to centrifugation at 700 × *g* at 4 °C for 2.5 h using a P40ST rotor (HITACHI). Following centrifugation, the fractions were monitored using an OD_254nm_ ultraviolet spectrometer. For immunoblotting analysis, the fractions were mixed with SDS sample buffer.

### 2.24 Nascent protein detection

We employed Click-iT reagents (Invitrogen) to detect nascent proteins. Cells were cultured in methionine-free DMEM (GIBCO) for 30 min before the introduction of l-homopropargylglycine (HPG), a methionine analog. As a negative control, we added 0.353 mmol/L of cycloheximide, a protein synthesis inhibitor, 30 min before HPG treatment. Following the Click-iT reaction, the cells underwent immunofluorescence analysis.

### 2.25 Wound-healing assay

VSMCs were seeded in 6-well plates at appropriate densities. Once confluent, each well was scratched using a 10 μL pipette tip. After scratching, wells were washed with PBS to remove detached cells, and complete culture media was added for further incubation. Wound healing was recorded under a microscope at 12 and 24 h post-scratching.

### 2.26 Cell counting kit-8 (CCK-8) assay

VSMCs were seeded into a 96-well plate at the same density and incubated with serum-free medium for 24 h. Subsequently, complete medium with appropriate stimuli was added, and the cells were further incubated for 24–48 h. After that, the culture medium in the 96-well plate was removed, and 100 μL of fresh medium containing 10% CCK-8 reagent was added. The cells were then incubated for an additional 4 h, followed by measurement of the absorbance at 450 nm using a microplate reader. This absorbance value reflects the relative quantity of cells.

### 2.27 Statistical analysis

Data are expressed as mean ± standard deviation (SD), and statistical analysis was performed using GraphPad Prism (version 8; GraphPad Software, San Diego, CA, USA). Normality was checked using the Shapiro–Wilk test. For comparisons between 2 groups, a two-tailed unpaired Student’s *t*-test was used when the data passed the normality test; otherwise, the Mann–Whitney U-test with exact method was used. One-way analysis of variance (ANOVA) followed by Dunnett’s or Tukey’s multiple comparisons tests or two-way ANOVA with Tukey’s multiple comparisons test was used with adjustment for multiplicity for comparison between three or more groups that passed the normality test. The correlation coefficient *r* was calculated using Spearman’s correlation coefficient test. Statistical significance was set at *P* < 0.05. Each data point is plotted individually on the graphs, and the exact *P*-values are stated.

### 2.28 Data availability

All data associated with this study are present in the paper or the Supporting Information RNA sequencing data have been deposited into the GEO database (www.ncbi.nlm.nih.gov/geo/) under accession number GSE234349. The mass spectrometry data for the identification of NFATc3-binding proteins in MASMCs (ProteomeXchange ID: PXD042816) have been deposited into the iProX database (http://www.iprox.org). The transcriptomic sequencing data have been deposited into the National Center for Biotechnology Information GEO and are accessible through the GEO series accession number GSE234349. The data of this study can also be obtained from the corresponding author upon reasonable request.

## 3 Results

### 3.1 NFATc3 is upregulated in human and mouse AAD

**Figure 1:**
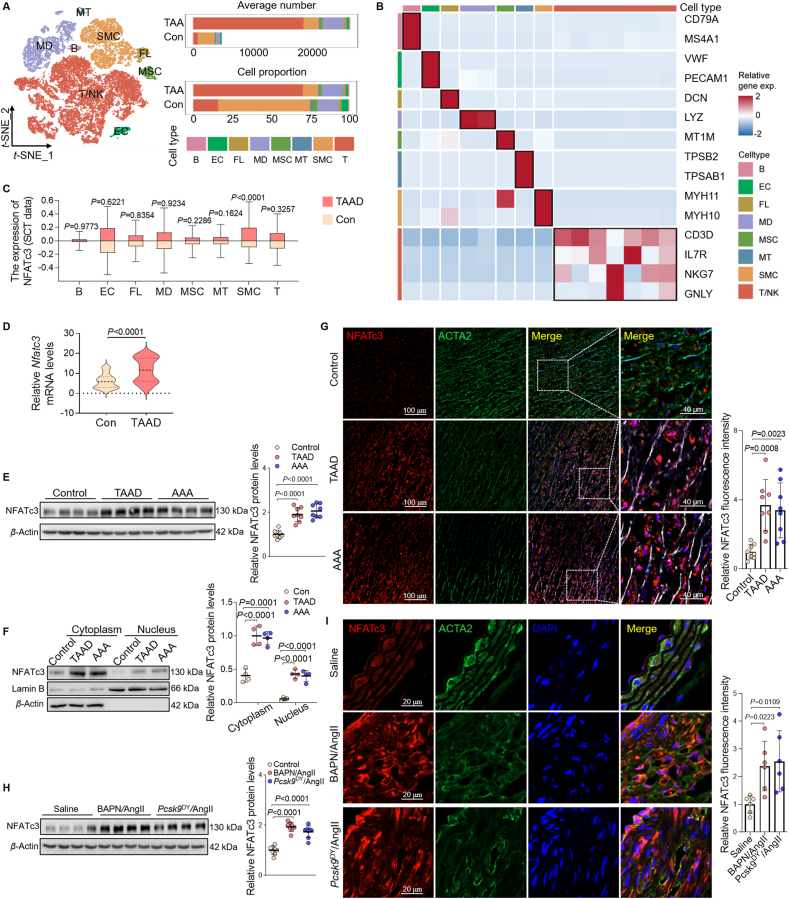
NFATc3 is upregulated during human and mouse AAD. (A) *t*-Distributed stochastic neighbor embedding (*t*-SNE) visualization of cell types isolated from human ascending aortic wall tissue *via* scRNA-seq (GSE155468). (B) Mean expression values scaled *via* mean-centering and transformed to a scale from −2 to 2. (C) Boxplot of NFATc3 expression level across major cell types. (D) Relative *NFATc3* mRNA in the ascending aortas of patients undergoing coronary bypass, but without AAD (Con), and patients with TAAD. *n* = 36. (E) Relative NFATc3 protein levels in ascending aortas of patients with or without TAAD, and in abdominal aortas of patients with AAA (*n* = 8). (F) Western blot analysis of NFATc3 levels in the cytoplasm and nucleus of aortas from patients without TAAD and those with TAAD or AAA (*n* = 4). (G) NFATc3 and ACTA2 immunofluorescence in aortas of patients without TAAD or with TAAD or AAA (*n* = 8). (H) Relative NFATc3 protein levels in mice aortas treated with saline, BAPN/AngII, or AAV-*Pcsk9*^DY^/AngII (*n* = 8). (I) NFATc3 and ACTA2 immunofluorescence in mice aortas treated with saline, BAPN/AngII, or AAV-*Pcsk9*^DY^/AngII (*n* = 6). Data are presented as mean ± SD. (C, D) Mann–Whitney U-test with the exact method; two-tailed *P*-values; (E–I) One-way ANOVA followed by Dunnett’s correction; adjusted *P*-values.

To investigate whether NFATc3 expression is associated with aortic aneurysm, we re-analyzed the single-cell RNA sequencing GEO database (GSE155468) generated from aortic tissue from patients with healthy ascending aortic tissue or diagnosed with TAA. We annotated 8 major cell types based on classical cell markers (Fig. 1A and B). The random sampling analysis suggested that NFATc3 expression was significantly upregulated in SMCs within the TAA group (Fig. 1C). Consistent with the GEO database results, NFATc3 mRNA levels increased in human TAAD tissues obtained from patients undergoing ascending aorta replacement surgery. Control aortic tissues were obtained *via* coronary bypass (Fig. 1D). NFATc3 protein levels increased within the nuclear and cytoplasmic fractions of human TAAD and AAA tissues (Fig. 1E and F). Immunofluorescence revealed that human TAAD and AAA lesions exhibited decreased ACTA2 and increased NFATc3 expression, compared to control aortic tissues (Fig. 1G).

Two animal models were developed: an AAD model created through BAPN/AngII treatment^16^ and an AAA model created by inducing hyperlipidemia plus AngII infusion^17^ (Supporting Information Fig. S1A and S1B). We used an AAV encoding *Pcsk9* D377Y (Asp374-to-Tyr mutant) to induce hyperlipidemia^15^^,^^17^. NFATc3 protein levels significantly increased in BAPN/AngII-induced AAD tissues and *Pcsk9*^DY^/AngII-induced AAA tissues (Fig. 1H). Immunofluorescence of aortic tissues revealed that mice treated with BAPN/AngII or *Pcsk9*^DY^/AngII exhibited decreased ACTA2 and increased NFATc3 expression, compared to saline-treated aortic tissues (Fig. 1I). Hence, NFATc3 may play a role in AAD.

### 3.2 NFAT3 deletion in VSMC mitigates AAD in mice

**Figure 2:**
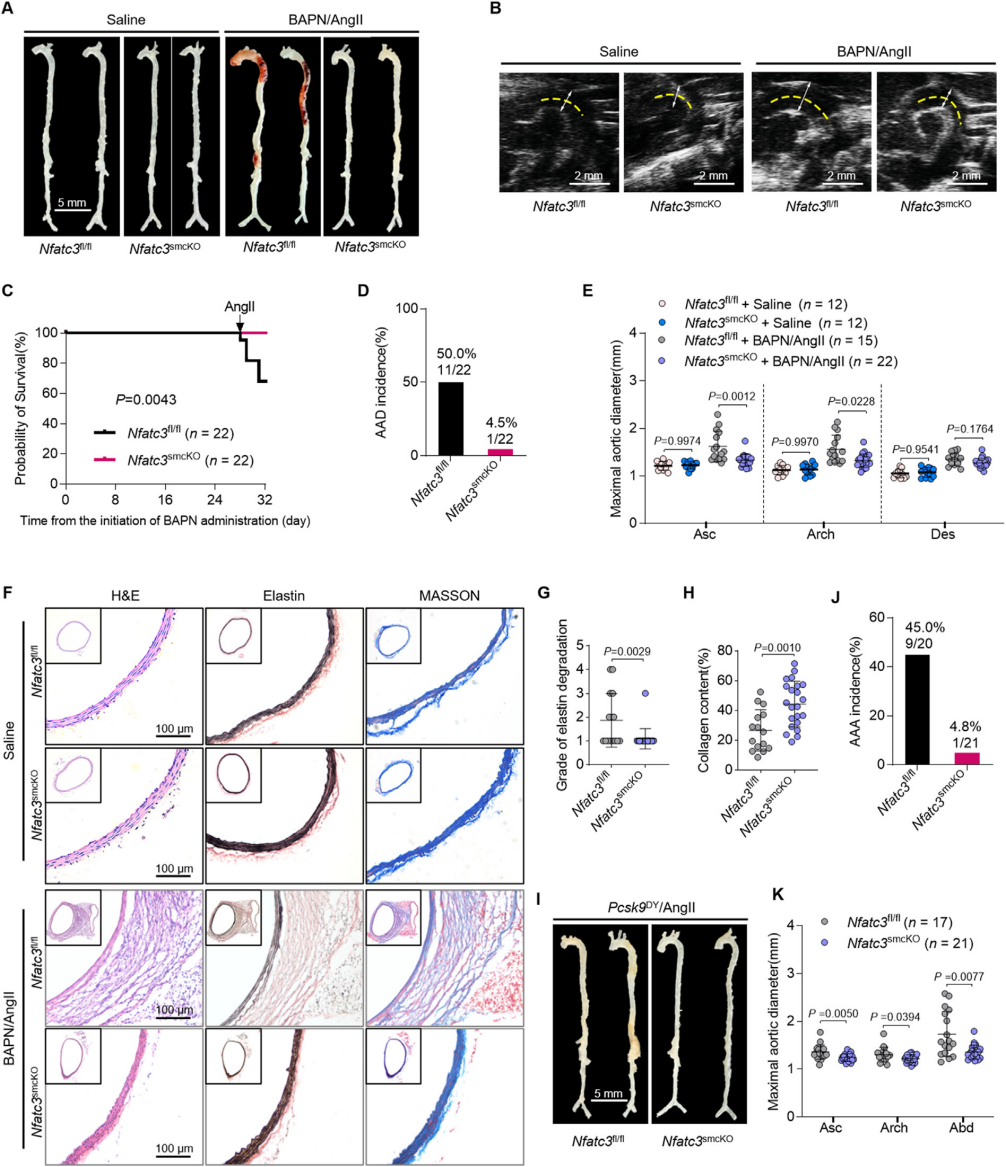
VSMC–NFATc3 deficiency mitigates AAD in mice. (A–H) Five-week-old *Nfatc3*^fl/fl^ or *Nfatc3*^smcKO^ male mice administered BAPN (0.3 g/kg/day) for 28 days, followed by AngII infusion (1000 ng/kg/min) for 3 days (*n* = 22). (A) Mouse aorta macrographs. (B) Thoracic aorta ultrasound images. (C) Survival curves analyzed *via* Kaplan–Meier and compared using log-rank tests. (D) AAD incidence per group. (E) Maximal aortic diameter *ex vivo* (*n* = 15 for *Nfatc3*^fl/fl^; *n* = 22 for *Nfatc3*^smcKO^). (F) H&E, van Geison (elastin), and Masson staining of mouse ascending aorta. (G, H) Elastin degradation grade (G) and collagen content (H) in the aortic wall. (I–K) Eight-week-old *Nfatc3*^fl/fl^ or *Nfatc3*^smcKO^ mice were injected AAV-*Pcsk9*^DY^*via* the tail vein and fed a Western-type diet. The mice were infused with AngII (1500 ng/kg/min) after 2 weeks for another 4 weeks. (I) Mouse aorta macrographs. (J) AAA incidence per group. (K) Maximal aortic diameter *ex vivo* (*n* = 17 for *Nfatc3*^fl/fl^; *n* = 21 for *Nfatc3*^smcKO^). Mice that died from aortic rupture were not included in the measurements. Data are presented as mean ± SD. (E, H, K) Unpaired Student’s *t*-test; two-tailed *P*-values. (G) Mann–Whitney U-test with the exact method; two-tailed *P*-values.

We created VSMC-NFATc3-knockout mice (*Myh11-*creER^T2^-Cre/*Nfatc3*^fl/fl^), also known as *Nfatc3*^smcKO^ mice (Fig. S1C and S1D). The null allele was confirmed in VSMCs from *Nfat3*^smcKO^ mice using immunofluorescence and Western blot (Fig. S1E and S1F). In *Nfatc3*^fl/fl^ and *Nfatc3*^smcKO^ mice, we created a BAPN/AngII-induced AAD model (Fig. S1G). No difference was observed in aortic morphology between saline-infused *Nfatc3*^smcKO^ and *Nfatc3*^fl/fl^ mice (Fig. 2A). With BAPN/AngII treatment, NFATc3 deletion in VSMCs prevented AAD development, with lower mortality rates and AAD incidence than those in *Nfatc3*^fl/fl^ mice (Fig. 2A–D). *Nfatc3*^smcKO^ mice exhibited a significant decrease in the maximum aortic diameter compared to *Nfatc3*^fl/fl^ mice (Fig. 2E). VSMC–NFATc3 knockout decreased elastin and collagen degradation (Fig. 2F–H), whereas body weight and plasma lipid levels were comparable in *Nfatc3*^fl/fl^ and *Nfatc3*^smcKO^ mice treated with saline or BAPN/AngII (Fig. S1H–S1L). However, *Nfatc3*^smcKO^ mice exhibited decreased blood pressure during AngII infusion (Fig. S1M).

A *Pcsk9*^DY^/AngII-induced AAA model was created in *Nfatc3*^fl/fl^ and *Nfatc3*^smcKO^ mice (Supporting Information Fig. S2A). The *Pcsk9*^DY^ mutation (encoding the Asp374-to-Tyr mutant) induces the degradation of hepatic low-density lipoprotein receptors (LDLR) in hepatocytes, closely mimicking the phenotype observed in *LDLR*^−/−^ mice and effectively induces hyperlipidemia^18^. Expectedly, LDLR protein level was almost undetectable, while PCSK9 was significantly overexpressed in the liver following AAV-*Pcsk9*^DY^ infection (Fig. S2B). *Nfatc3*^smcKO^ mice exhibited significantly reduced AAA development (Fig. 2I) and a lower mortality rate, AAA incidence, and maximal abdominal aorta diameters than *Nfatc3*^fl/fl^ mice (Fig. 2J and K, Fig. S2C and S2D). *Nfatc3*^smcKO^ mice also presented with decreased elastin degradation, increased collagen content (Fig. S2E–S2G), and decreased blood pressure following AngII infusion (Fig. S2H). However, no significant difference in body weight, total cholesterol (TC), or plasma triglyceride (TG) levels was observed between *Nfatc3*^fl/fl^ and *Nfatc3*^smcKO^ mice (Fig. S2I–S2K). These findings indicate a beneficial role of VSMC–NFATc3 deficiency in AAD development.

### 3.3 VSMC–NFATc3 overexpression aggravates AAD in mice

We used CRISPR/Cas9-based *Rosa26* insertion with a conditional inducible Cre/Loxp to create a mouse strain carrying the human-*NFATc3* knock-in allele to verify the VSMC–NFATc3 function in AAD development. The mice were then crossed with Myh11-cre/ER^T2^ transgenic mice to obtain VSMC–NFATc3 knock-in mice (*Myh11-*cre*ER*^T2^-Cre/*Rosa26-NFATc3*), designated *Nfatc3*^smcKI^ mice (Supporting Information Fig. S3A). After tamoxifen administration, we examined NFATc3 expression in *Nfatc3*^smcKI^ mice. A *Rosa26-NFATc3* homozygote with *Myh11*-cre and a *Rosa26-NFATc3* homozygote without *Myh11*-cre (*Nfatc3*-KI^fl/fl^ mice) from mouse tail DNA were detected (Fig. S3B). Furthermore, NFATc3 levels in VSMCs were significantly higher in *Nfatc3*^smcKI^ than in *Nfatc3*-KI^fl/fl^ mice (Fig. S3C and S3D).

**Figure 3:**
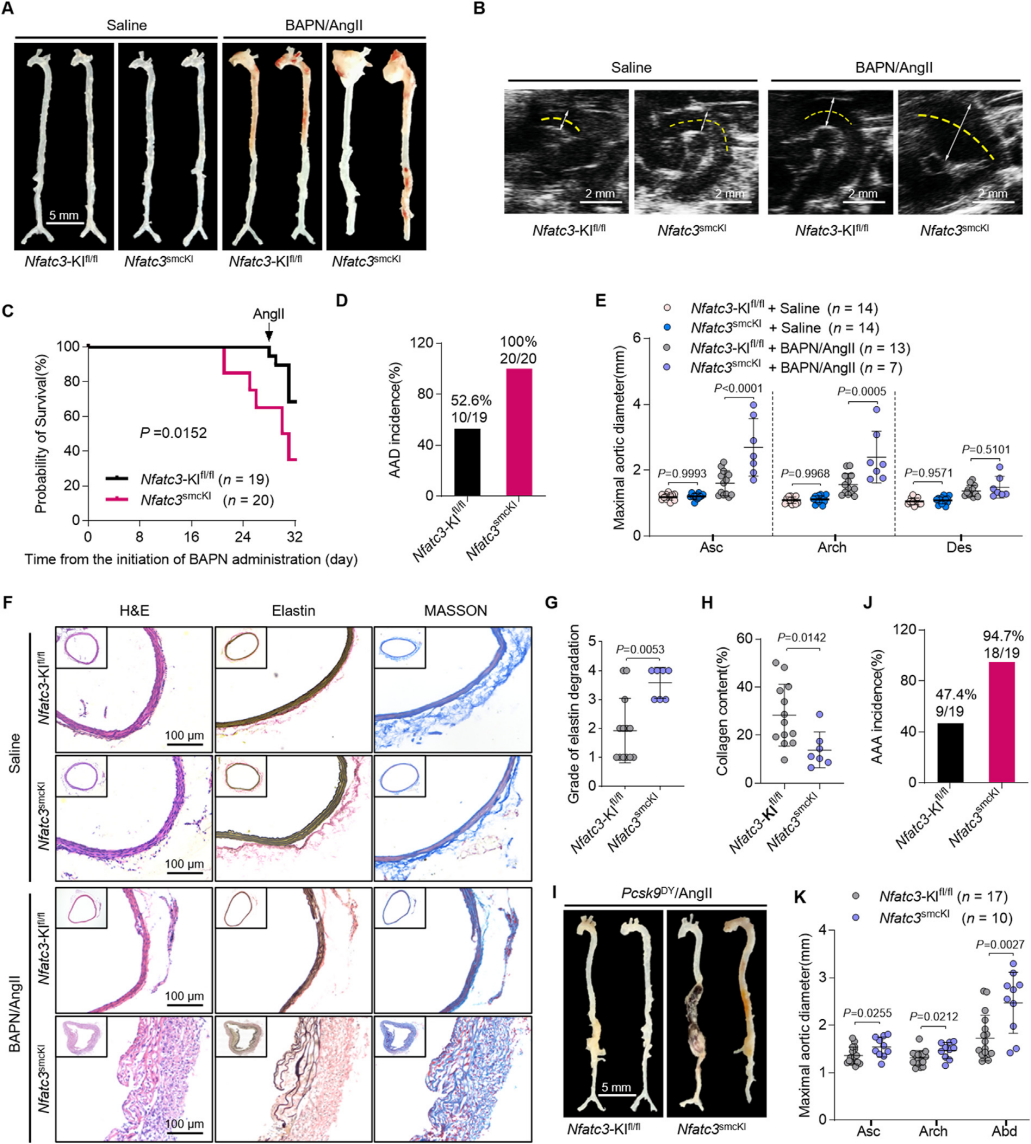
VSMC–NFATc3 overexpression aggravates AAD in mice. (A–H) Five-week-old *Nfatc3*-KI^fl/fl^ or *Nfatc3*^smcKI^ male mice were administered BAPN (0.3 g/kg/day) for 28 d before infusion with AngII (1000 ng/kg/min) for 3 d (*n* = 19 for *Nfatc3*-KI^fl/fl^; *n* = 20 for *Nfatc3*^smcKI^). (A) Mouse aorta macrographs. (B) Thoracic aorta ultrasound images. (C) Survival curves per group analyzed using Kaplan–Meier and compared using log-rank tests. (D) AAD incidence. (E) Maximal aortic diameter *ex vivo* (*n* = 13 for *Nfatc3*-KI^fl/fl^; *n* = 7 for *Nfatc3*^smcKI^). (F) H&E, van Geison (elastin), and Masson staining of the mouse ascending aorta. (G, H) Elastin degradation grade (G) and collagen content (H) in the aortic wall. (I–K) Eight-week-old *Nfatc3*-KI^fl/fl^ or *Nfatc3*^smcKI^ mice injected AAV-*Pcsk9*^DY^*via* tail vein and fed a Western-type diet. Two weeks later, the mice were infused with AngII (1500 ng/kg/min) for 4 weeks. (I) Mouse aorta macrographs. (J) AAA incidence. (K) Maximal aortic diameter *ex vivo* (*n* = 13 for *Nfatc3*-KI^fl/fl^; *n* = 10 for *Nfatc3*^smcKI^). Mice that died from aortic rupture were not included in the measurements. Data are presented as mean ± SD. (E, H) Unpaired Student’s *t*-test; two-tailed *P*-values. (G, K) Mann–Whitney U-test with the exact method; two-tailed *P*-values.

We constructed BAPN/AngII-induced AAD and AAV-*Pcsk9*^DY^/AngII-induced AAA models to investigate the role of VSMC–NFATc3 overexpression in aortic degeneration, dissection, and rupture (Fig. S3E, Supporting Information Fig. S4A). The aortic morphology of saline-infused *Nfatc3*-KI^fl/fl^ and *Nfatc3*^smcKI^ mice was comparable (Fig. 3A). VSMC–NFATc3 overexpression aggravated AAD during BAPN/AngII treatment, with a higher mortality rate and 100% AAD incidence than those in *Nfatc3*-KI^fl/fl^ mice (Fig. 3B–D). Compared with *Nfatc3*-KI^fl/fl^ mice, *Nfatc3*^smcKI^ mice exhibited a significant increase in maximum aortic diameter (Fig. 3E). Moreover, VSMC–NFATc3 overexpression increased elastin and collagen degradation (Fig. 3F–H). However, the *Nfatc3*^smcKI^ mice body weight, plasma TC, and TG levels did not differ from those of *Nfatc3*-KI^fl/fl^ mice administered saline or BAPN/AngII (Fig. S3F–S3H). AngII infusion elevated blood pressure in *Nfatc3*^smcKI^ mice (Fig. S3I).

An AAV-*Pcsk9*^DY^/AngII-induced AAA model was established in *Nfatc3*-KI^fl/fl^ and *Nfatc3*^smcKI^ mice (Fig. S4A). VSMC–NFATc3 overexpression aggravated AAA, with increased mortality rate, higher incidence, larger abdominal aorta maximal diameter, increased elastin degradation, and decreased collagen content (Fig. 3I–K, Fig. S4B–S4F). However, body weight, plasma TC, and TG levels did not differ between groups (Fig. S4G–S4I). Nonetheless, AngII infusion elevated blood pressure in *Nfatc3*^smcKI^ mice (Fig. S4J). These findings suggest that VSMC–NFATc3 aggravates AAA development.

### 3.4 VSMC–NFATc3 accelerates TAAD in the BAPN model

VSMC–NFATc3 overexpression elevated systolic blood pressure following AngII infusion. However, VSMC–NFATc3 overexpression aggravated mouse mortality induced by AAD progression during a BAPN-fed period without AngII infusion (Fig. 3C).

**Figure 4:**
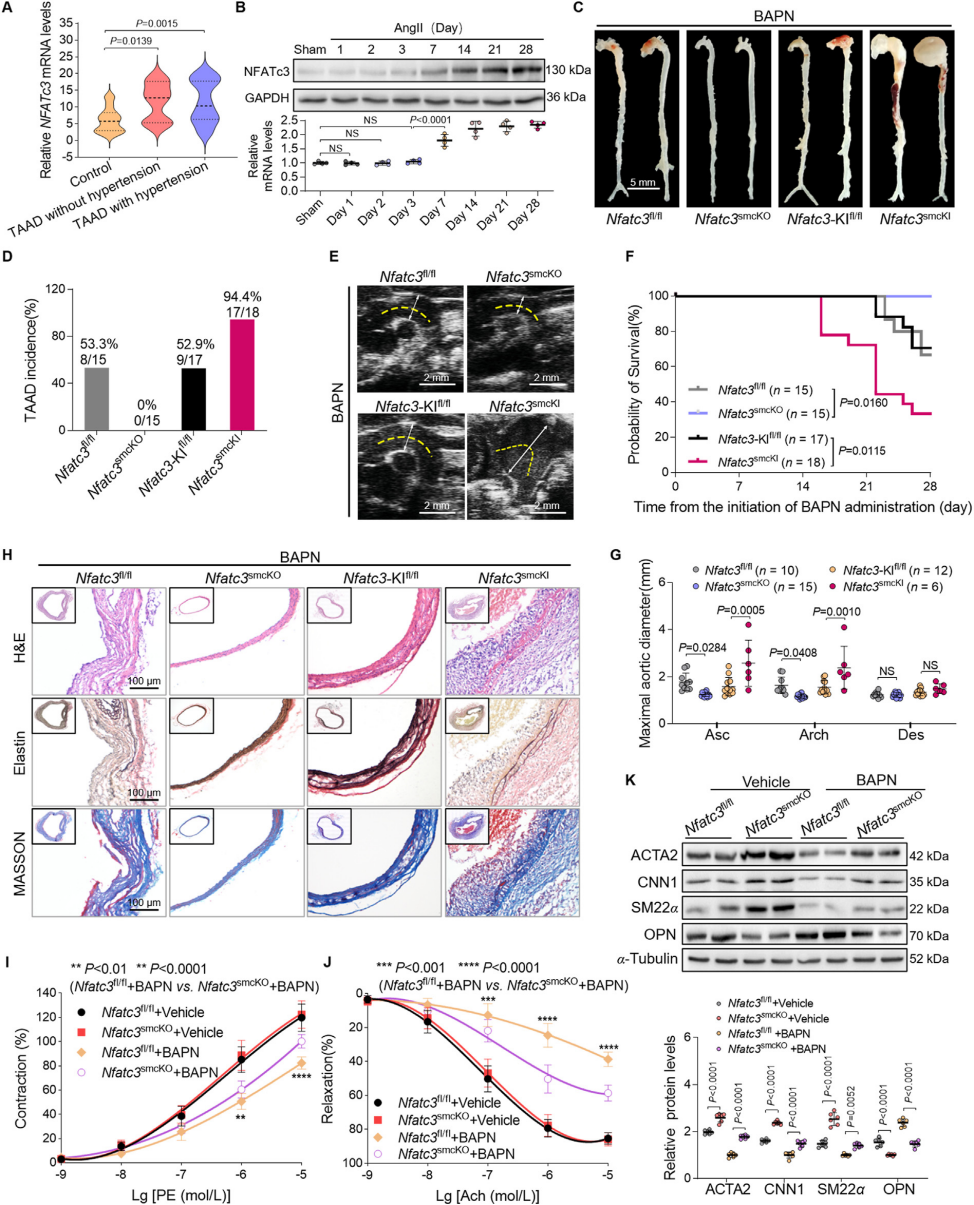
VSMC–NFATc3 facilitates AAD progression in the BAPN model. (A) Relative NFATc3 mRNA levels in ascending aortas of patients without TAAD undergoing coronary bypass (Control, *n* = 36) and TAAD with and without hypertension (*n* = 25; 11, respectively). (B) NFATc3 Western blot in mouse aortas with different AngII treatment durations (*n* = 4). (C–I) Five-week-old male *Nfatc3*^fl/fl^ (*n* = 15), *Nfatc3*^smcKO^ (*n* = 15), *Nfatc3*-KI^fl/fl^ (*n* = 17), and *Nfatc3*^smcKI^ (*n* = 18) mice were administered BAPN (0.6 g/kg/day) for 28 days. (C) Aorta macrographs. (D) TAAD incidence. (E) Thoracic aorta ultrasound images. (F) The survival rate was estimated using Kaplan–Meier and compared using the log-rank test. (G) Maximum aortic diameter. (H) H&E, elastin, and Masson aorta staining. (I, J) Phenylephrine (PE)-induced vascular contraction (I) and acetylcholine (Ach)-induced endothelium-dependent vasodilation (J) of aortic rings from *Nfatc3*^fl/fl^ and *Nfatc3*^smcKO^ mice treated with vehicle or BAPN for 28 days (*n* = 8). (K) Western blot and ACTA2, CNN1, SM22*α*, and OPN quantification in aortas (*n* = 6). Mice that died from aortic rupture were not included in the measurements. Data are presented as mean ± SD. (A, B, G) One-way ANOVA with Tukey’s correction; adjusted *P*-values. (I–K) Two-way ANOVA with Tukey’s correction; adjusted *P*-values. NS, no significance (*P* > 0.05).

We also investigated whether NFATc3 expression levels in patient TAAD tissues correlated with blood pressure to determine the mechanism of NFATc3-related AAD. Notably, compared to that in controls, NFATc3 expression was increased in patients with TAAD without hypertension (Fig. 4A). To explore the underlying mechanisms linking NFATc3 to blood pressure and AAD development, NFATc3 expression was assessed in the mouse aorta under different durations of AngII infusion. No changes in NFATc3 expression were observed until the third day of AngII infusion (Fig. 4B, Supporting Information Fig. S5A). These findings suggest that elevated NFATc3 expression promotes AAD development *via* a potential blood pressure-independent mechanism and that abnormal NFATc3 expression is critical for AAD.

Considering that BAPN can induce TAAD in mice independent of AngII^19^^,^^20^, we used another AAD model with 4-week BAPN treatment to verify the role of VSMC–NFATc3 in TAAD (Fig. S5B). VSMC–NFATc3 had a negligible effect on blood pressure, body weight, plasma TC, and TG levels (Fig. S5C–S5F). VSMC–NFATc3 deficiency in mice significantly lowered BAPN-induced mortality, TAAD incidence, and aortic dilation, whereas VSMC-NFATc3-overexpressing mice exhibited the opposite phenotype (Fig. 4C–G). NFATc3 accelerated elastic fiber fragmentation and disarray, increased collagen degradation, and promoted medial degeneration (Fig. 4H, Fig. S5G and S5H). VSMC–NFATc3 deficiency affected the systolic and diastolic functions of blood vessels (Fig. 4I and J). Moreover, VSMC–NFATc3 deletion affected the VSMC phenotypic switch *in vivo*. Following BAPN administration, *Nfatc3*^fl/fl^ mice exhibited a significant decrease in ACTA2, CNN1, and SM22*α* and an increase in osteopontin (OPN) expression in the aorta, whereas VSMC–NFATc3 deletion recovered contractile marker levels and decreased OPN expression (Fig. 4K). Since TAAD is closely associated with inflammation, we next investigated the effect of VSMC-NFATc3 on the expression of major inflammatory factors in the aortic wall. Under BAPN or AAV-*Pcsk9*^DY^/AngII induction, NFATc3 did not affect the expression of inflammatory cytokines such as IL-6, IL-1*β*, and TNF-*α* in the in VSMCs of the aortic wall (Supporting Information Fig. S6A and S6B). These results confirmed that NFATc3 deficiency in VSMCs facilitates the phenotypic switch in TAAD pathogenesis without affecting the expression levels of inflammatory cytokines in TAAD.

### 3.5 Nuclear NFATc3 promotes MMP activation and ECM degradation

**Figure 5:**
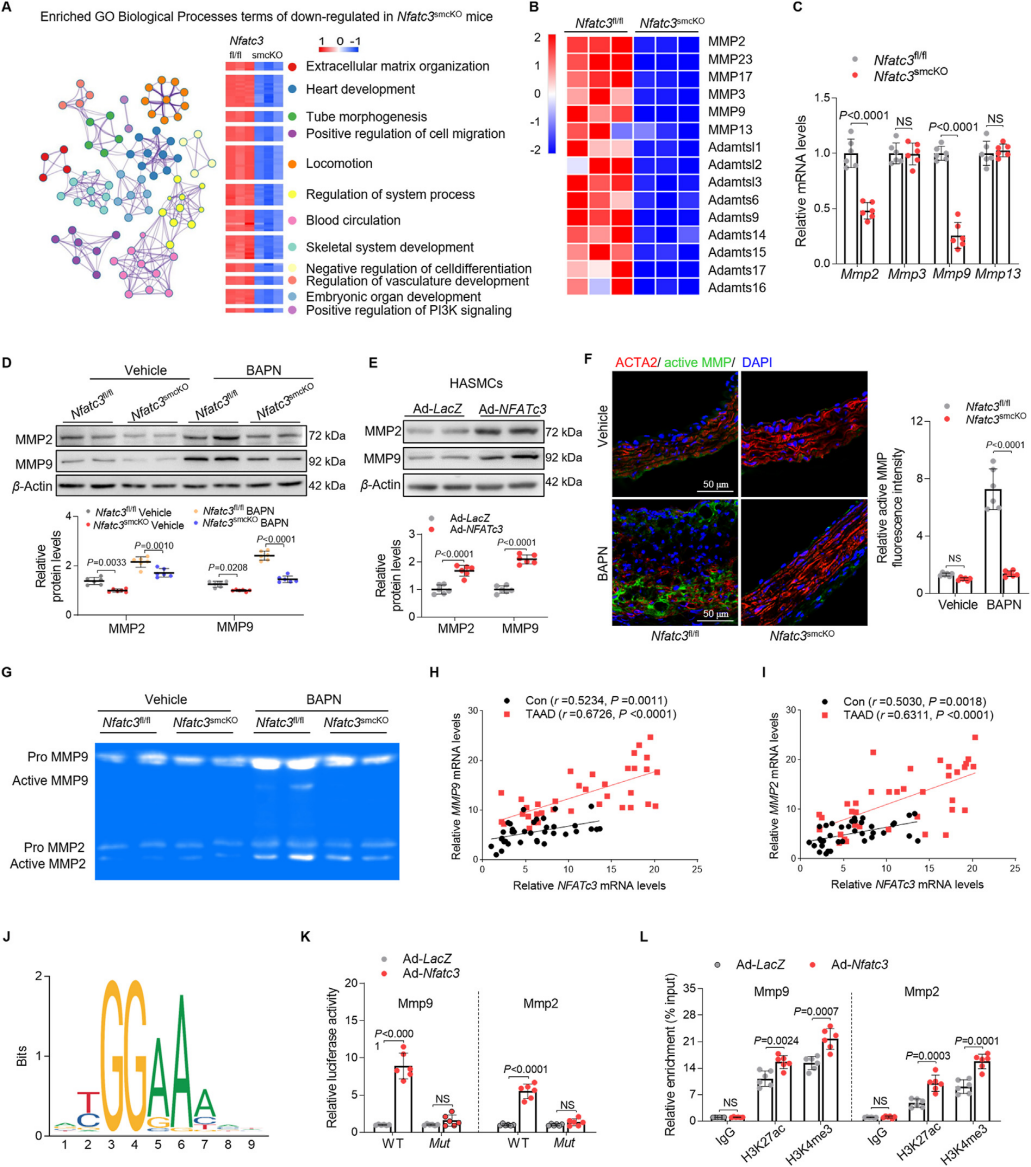
NFATc3 promotes ECM degradation by transcriptionally upregulating MMP9 and MMP2 expression in VSMCs. (A) Gene Ontology term enrichment analysis of the biological process influenced by NFATc3 deficiency based on the RNA-seq dataset. Downregulated processes in the aortas of *Nfatc3*^smcKO^ mice compared to those in *Nfatc3*^fl/fl^ mice treated with BAPN (0.6 g/kg/day) for 28 days (*n* = 3). (B) Gene expression profiles were compared between *Nfatc3*^smcKO^ to *Nfatc3*^fl/fl^ mice treated with BAPN, and heatmaps were generated based on the expression of significantly different genes related to ECM organization (blue, downregulated; red, upregulated). Genes with a corresponding adjusted *P* < 0.05 were considered statistically significant. (C) Relative mRNA levels of genes related to ECM organization in the aortas of *Nfatc3*^smcKO^ and *Nfatc3*^fl/fl^ mice treated with BAPN (*n* = 6). (D) MMP2 and MMP9 Western blot in the aortas of *Nfatc3*^smcKO^ and *Nfatc3*^fl/fl^ mice treated with vehicle or BAPN (*n* = 6). (E) MMP2 and MMP9 protein levels in HASMCs infected with Ad-*LacZ* and Ad-*NFATc3* (*n* = 6). (F) Immunofluorescence of *in situ* zymography and immunostaining for ACTA2 in *Nfatc3*^fl/fl^ and *Nfatc3*^smcKO^ ascending aortas after vehicle or BAPN treatment (*n* = 6). (G) Gelatin zymography for detecting pro-MMP9, active-MMP9, pro-MMP2, and active-MMP2 in the ascending aortas of *Nfatc3*^fl/fl^ and *Nfatc3*^smcKO^ mice after vehicle or BAPN treatment. (H, I) Correlation analysis of *NFATc3* mRNA with *MMP9* (H) or *MMP2* (I) levels in the ascending aortas of patients without AAD (Con) (*n* = 36) and with TAAD (*n* = 36). (J) NFATc3-binding motifs. (K) Luciferase reporter analysis in 293T cells (*n* = 6). (L) ChIP-qPCR validation of H3K27ac and H3K4me3 enrichment on the *Mmp9* and *Mmp2* promoter (*n* = 6). Data are presented as mean ± SD. (C, E, L) Unpaired Student’s *t*-test; two-tailed *P*-values. (D, F, K) Two-way ANOVA with Tukey’s correction; adjusted *P*-values. (H, I) Spearman’s or Pearson’s correlation coefficient test; regression coefficients. NS, no significance (*P* > 0.05).

We performed high-throughput RNA-seq of mouse aortas with BAPN treatment to elucidate the underlying mechanisms linking NFATc3 to AAD etiology. Bioinformatics analyses of differentially expressed genes revealed downregulated genes, particularly in ECM organization (Fig. 5A, Supporting Information Fig. S7A and S7B). Structural ECM disruption and degradation caused MMP activation, particularly MMP2 and MMP9, a distinguishing hallmark of aortic aneurysm and rupture^21^^,^^22^. VSMC–NFATc3 deficiency significantly decreased *MMP* gene expression during ECM degradation and AAD development (Fig. 5B). *NFATc3* knockout reduced *Mmp9* and *Mmp2* mRNA expression (Fig. 5C), whereas BAPN increased MMP2 and MMP9 expression (Fig. 5D, Fig. S7C). NFATc3 deletion/overexpression decreased and increased MMP9 and MMP2 expression, respectively, in the aortas (Fig. 5D, Fig. S7C). Compared to those in the controls, MMP9 and MMP2 mRNA and protein levels increased in Ad-NFATc3-treated HASMCs (Fig. 5E, Fig. S7D). MMP activities were consistently reduced in ascending aortic sections from *Nfatc3*^smcKO^ mice compared to those in *Nfatc3*^fl/fl^ mice after BAPN treatment. Conversely, the ascending aortic sections from *Nfatc3*^smcKI^ mice exhibited increased MMP activity (Fig. 5F, Fig. S7E). Similarly, gelatin zymography revealed that MMP9 and MMP2 activity in the *Nfatc3*^smcKO^ mouse aortas decreased significantly (Fig. 5G).

NFATc3 levels in human aortas correlated with *MMP9* and *MMP2* levels in normal controls and patients with TAAD (Fig. 5H and I), suggesting that NFATc3 regulates MMP9 and MMP2 expression. This transcriptional regulation in VSMCs was investigated further. Nuclear run-on experiments revealed that NFATc3 upregulated *MMP9* and *MMP2* transcript levels (Fig. S7F and S7G). Hence, potential NFATc3-binding sites in the *MMP9* and *MMP2* promoters (−1.0–0.5 and −2.0–1.5 kb fragments upstream of the transcription start site, respectively) were searched using the JASPER database (Fig. 5J). The functional NFATc3 site was centered on a conserved evolutionary site (Fig. S7H and S7I). Enrichment at *MMP9* and *MMP2* promoter fragment sites differed from that at the IgG control (Fig. S7J and S7K). NFATc3 increased *Mmp9* and *Mmp2* promoter-driven luciferase activity, and mutation of the putative NFATc3 binding site diminished the NFATc3-dependent effect (Fig. 5K). Reciprocally, NFATc3 enriched H3K27ac (enhancer and promoter marker) and H3K4me3 (promoter-specific marker) on the proximal *Mmp9* and *Mmp2* promoters in VSMCs (Fig. 5L).

We also assessed and quantified fibronectin and COL1 expression in the aortic lesion area. Fibronectin and COL1 protein expression increased in *Nfatc3*^smcKO^ mouse lesions after BAPN treatment (Fig. S7L). Immunofluorescence staining of aortic sections revealed significantly higher fibronectin and COL1 density in the dissection area of *Nfatc3*^smcKO^ mice than in that of *Nfatc3*^fl/fl^ mice after BAPN treatment (Fig. S7M and S7N). Therefore, VSMC–NFATc3 promotes MMP activation and ECM degradation, and NFATc3 directly targets and transcriptionally upregulates MMP9 and MMP2 levels.

### 3.6 Cytoplasmic NFATc3 exacerbates the VSMC synthetic phenotype by facilitating global protein synthesis in ribosomes

**Figure 6:**
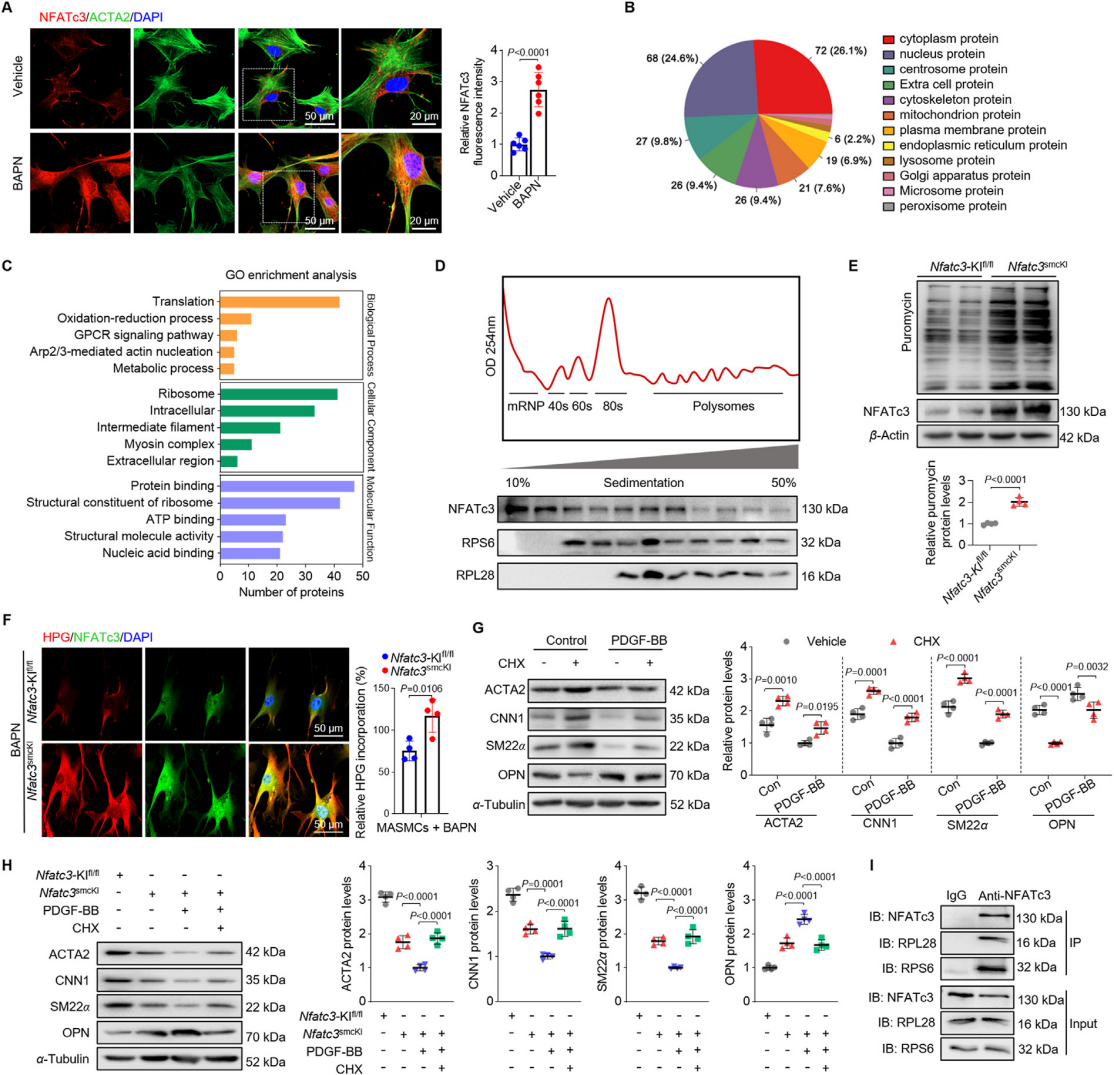
Cytoplasmic NFATc3 facilitates VSMC contractile-to-synthetic phenotype switching by promoting global protein synthesis. (A) Representative images and quantification of NFATc3 and ACTA2 expression using immunofluorescence staining in MASMCs isolated from mice treated with vehicle and BAPN for 28 days (*n* = 6). (B) NFATc3-associated protein subcellular localization based on LC–MS/MS. (C) Gene Ontology analysis of NFATc3-associated proteins identified using LC–MS/MS. (D) Polysome profiling shows NFATc3 distribution in MASMCs using sucrose density gradient centrifugation (*n* = 3). Western blot of NFATc3 in the elution profiles. (E) Representative Western blot images and quantification of puromycin incorporation assays from MASMCs isolated from *Nfatc3*-KI^fl/fl^ and *Nfatc3*^smcKI^ mice (*n* = 4). (F) HPG incorporation assay in VSMCs isolated from the aortas of *Nfatc3*-KI^fl/fl^ and *Nfatc3*^smcKI^ mice treated with BAPN for 28 days (*n* = 4). (G) Western blot and quantification of ACTA2, CNN1, SM22*α*, and OPN in MASMCs treated with PDGF-BB for 24 h before a 12-h CHX treatment (*n* = 4). (H) Representative Western blot images and quantification of ACTA2, CNN1, SM22*α*, and OPN in MASMCs isolated from aortas of *Nfatc3*-KI^fl/fl^ and *Nfatc3*^smcKI^ mice and treated with PDGF-BB (20 μg/L) for 24 h before a 12-h CHX (50 μmol/L) treatment (*n* = 4). (I) Co-immunoprecipitation of MASMC lysates with anti-NFATc3 antibodies. Western blot of RPL28 and RPS6 levels. Data are presented as mean ± SD. (E) Unpaired Student’s *t*-test; two-tailed *P*-values. (G) Two-way ANOVA with Tukey’s correction; adjusted *P*-values. (H) One-way ANOVA with Tukey’s correction; adjusted *P*-values.

NFATc3 expression was increased in the nucleus and cytoplasm of VSMCs from the aortas during AAD progression (Fig. 1F–G and L). Immunofluorescence revealed that BAPN treatment increased NFATc3 levels in the nuclear and cytoplasmic fractions of mouse aortic smooth muscle cells (MASMCs; Fig. 6A). NFAT activation in platelets regulates platelet hemostatic and immune functions, suggesting its non-transcriptional roles in platelet pathophysiology^23^. This suggests that NFAT may have unnoticeable non-genomic roles even in nucleated cells, prompting us to investigate these roles.

We co-immunoprecipitated NFATc3-associated proteins from VSMCs from mouse aortas to investigate the physiological functions of cytoplasmic NFATc3 in VSMCs. Subcellular localization revealed that cytoplasmic proteins were the most abundant (Fig. 6B). Based on Gene Ontology enrichment analysis, NFATc3-associated proteins were significantly enriched in translation-ribosome-related proteins (Fig. 6C). We performed polysome profiling in MASMCs to confirm NFATc3 distribution in translational components. NFATc3 was primarily distributed in the mRNP, 40S, 60S, 80S, and polyribosome components (Fig. 6D). NFATc3 co-localized with translational components, suggesting its relation to translation machinery and its role in protein synthesis.

The role of NFATc3 during translation was also investigated by conducting a puromycin incorporation assay to monitor global protein synthesis in NFATc3-knockout and NFATc3-overexpressing VSMCs. NFATc3 overexpression and deficiency boosted and inhibited global protein synthesis in VSMCs compared to control cells, respectively (Fig. 6E, Supporting Information Fig. S8A).

VSMCs were treated with the amino acid analog HPG to detect nascent protein synthesis, followed by Click-iT^24^. NFATc3 overexpression and deficiency promoted and inhibited HPG incorporation into VSMCs, respectively, indicating that NFATc3 promotes the protein synthesis rate (Fig. 6F, Fig. S8B). Hence, NFATc3 deficiency impairs global protein synthesis in VSMCs.

We then investigated whether protein synthesis is related to VSMC phenotypic transition. Cycloheximide (CHX) increased VSMC contractile protein expression inhibited by platelet-derived growth factor-BB (PDGF-BB) (Fig. 6G). However, Y-320, which accelerates protein synthesis^25^, decreased VSMC contractile maker expression induced by TGF-*β* (Fig. S8C). Moreover, NFATc3 overexpression prevented PDGF-BB-induced VSMC contractile marker repression and elevated OPN expression, which CHX treatment reversed (Fig. 6H). NFATc3 deficiency increased VSMC contractile marker expression and OPN repression induced by TGF-*β*, which was reversed by Y-320 treatment (Fig. S8D). Downregulation of NFATc3 increased the PDGF-BB-induced VSMC contractile marker repression in HASMCs (Fig. S8E). Co-immunoprecipitation analysis revealed that NFATc3 interacted with RPL28 and RPS6, indicating that NFATc3 was directly located in the ribosome (Fig. 6I). Increased protein synthesis is often closely linked to cellular proliferation and migration. We evaluated the impact of NFATc3 on the proliferation and migration of VSMCs and found that the deletion of NFATc3 significantly decreased cell viability and proliferation rate, whereas overexpression of NFATc3 increased cell viability and proliferation rate (Fig. S8F and S8G). Therefore, cytoplasmic NFATc3 promotes VSMC contractile-to-synthetic phenotypic switching by facilitating translation and global protein synthesis in ribosomes.

### 3.7 NFATc3 facilitates global protein synthesis by suppressing eEF2 phosphorylation

**Figure 7:**
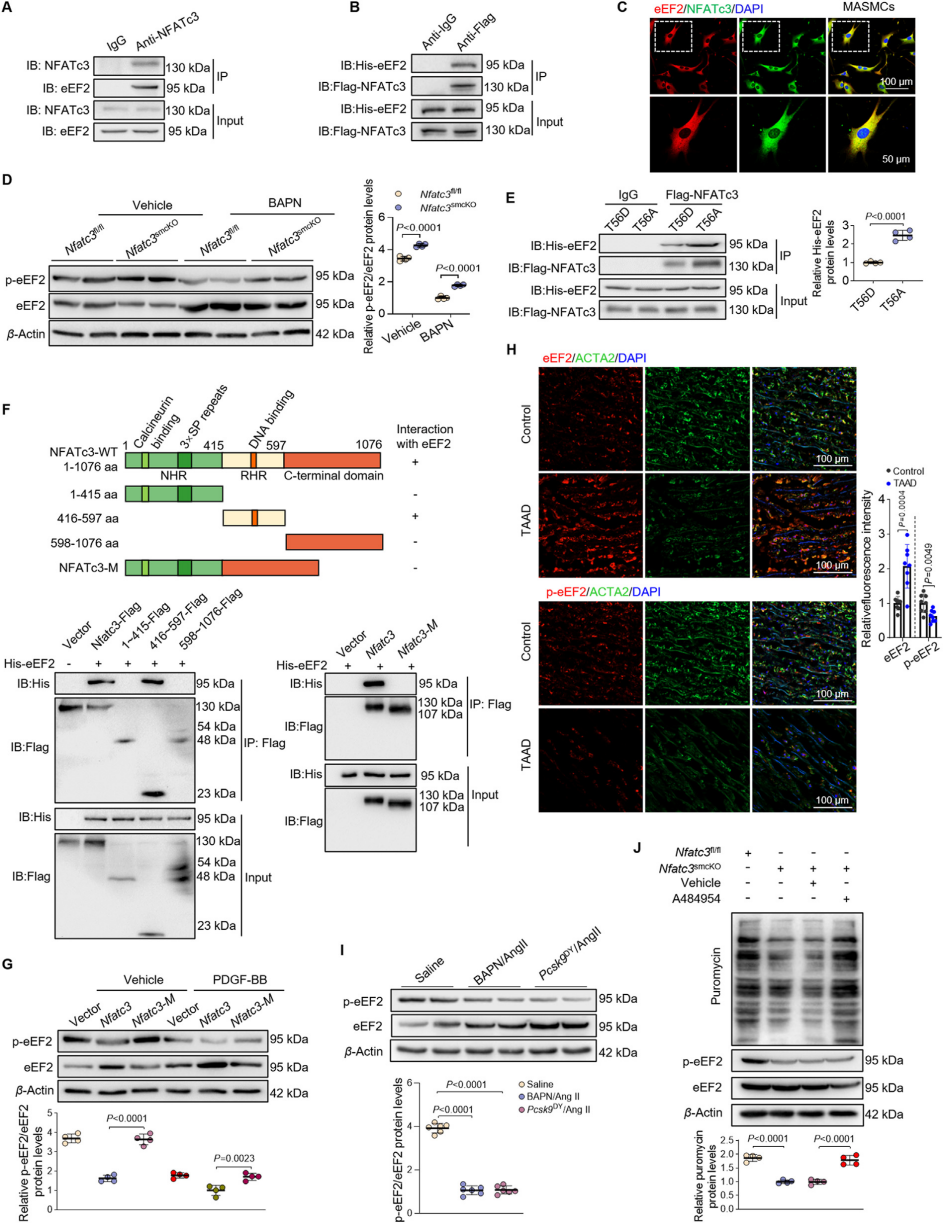
NFATc3 facilitates global protein synthesis by suppressing eEF2 phosphorylation. (A) Co-immunoprecipitation of MASMC lysates with anti-NFATc3 antibodies. The eEF2 level was determined using Western blot. (B) HEK293T cells were co-transfected with His-eEF2 and Flag-NFATc3 plasmids. Cell lysates were immunoprecipitated with anti-Flag antibodies, and the precipitates were analyzed using immunoblotting with anti-His antibodies. (C) Immunofluorescence for eEF2 and NFATc3 detection in MASMCs. (D) *Nfatc3*^fl/fl^ and *Nfatc3*^smcKO^ mice were treated with or without BAPN for 28 days. Western blot and quantification of p-eEF2 and eEF2 in aortic tissues (*n* = 4). (E) Western blots of binding assays show the binding preference of NFATc3. HEK293T cells were transfected with eEF2-T56D and eEF2-T56A plasmids for 48 h, and lysates were immunoprecipitated with anti-NFATc3 antibodies (*n* = 4). (F) Schematic illustration of NFATc3 structures and the presence/absence of binding between NFATc3 and eEF2 are indicated by + or – (top panel). Full-length His-eEF2 and various truncated Flag-NFATc3 forms were co-expressed in 293T cells, and immunoprecipitation with anti-Flag antibodies followed by Western blot (bottom panel) was performed. (G) Western blots and quantification of p-eEF2 and eEF2 in MASMCs transfected with vector, Plasmid-Nfatc3, or Plasmid-Nfatc3-Mutant before a 24-h PDGF-BB treatment (*n* = 4). (H) Representative images and quantification of eEF2 and ACTA2, p-eEF2, and ACTA2 expression using immunofluorescence staining in the ascending aortas of patients without TAAD and patients with TAAD (*n* = 8). (I) Relative eEF2 and p-eEF2 protein levels in the aortas of mice administered BAPN/AngII and AAV-*Pcsk9*^DY^/AngII (*n* = 6). (J) Western blot of puromycin incorporation assays and p-eEF2/eEF2 levels from MASMCs isolated from *Nfatc3*^fl/fl^ and *Nfatc3*^smcKO^ mice and treated with A484954 (10 μmol/L, 10 min) (*n* = 4). Data are presented as mean ± SD. (D) Two-way ANOVA with Tukey’s correction; adjusted *P*-values. (E, H) Unpaired Student’s *t*-test; two-tailed *P*-values. (G, I, J) One-way ANOVA followed by Tukey’s correction; adjusted *P*-values. NS, no significance (*P* > 0.05).

Considering that NFATc3 is critical for VSMC phenotype differentiation mediated by protein synthesis, we explored whether NFATc3 interacts with key proteins involved in translation elongation, particularly eEF2. The potential interaction between NFATc3 and eEF2 was validated. HASMCs were subjected to co-immunoprecipitation analysis, revealing an endogenous interaction between NFATc3 and eEF2 (Fig. 7A, Supporting Information Fig. S9A). Flag-NFATc3 and His-eEF2 plasmids were co-transfected into HEK293T cells for 48 h, whereas NFATc3 was co-immunoprecipitated with anti-His antibodies, and not IgG antibodies (Fig. 7B, Fig. S9B). Similarly, immunofluorescence confirmed an interaction between NFATc3 and eEF2 in MASMCs (Fig. 7C). NFATc3 was associated with non-phosphorylated eEF2, rather than p-eEF2 (Fig. S9C). However, while VSMC–NFATc3 deletion had little effect on the total eEF2 levels, it increased p-eEF2 levels in mouse aortas treated with vehicle or BAPN (Fig. 7D); NFATc3 did not affect eEF2K or PP2A expression levels (Fig. S9D and S9E). Hence, the elevated p-eEF2 levels were not due to eEF2K or PP2A activity changes. NFATc3 was directly localized in the ribosome, with a possible elongating function. We postulated that reduced NFATc3 binding to the eEF2 phosphorylation site caused changes in p-eEF2 levels.

We created two mutated plasmids expressing the eEF2^T56A^ and eEF2^T56D^ proteins to mimic non-phosphorylated eEF2 and p-eEF2 to determine whether NFATc3 binding blocked eEF2 phosphorylation. Expectedly, Flag-NFATc3 had a significantly higher affinity for non-phosphorylated eEF2^T56A^ than for eEF2^T56D^ (Fig. 7E), confirming that NFATc3 might bind directly to non-phosphorylated eEF2 around Thr56.

We subcloned full-length NFATc3 and three NFATc3 domains, including the NFAT-homology region (NHR, amino acids 1–415), REL-homology domain (RHR, amino acids 416–597), and C-terminal domain (amino acids 598–1076) into the pCMV6-Flag plasmid to investigate which NFATc3 domain binds to eEF2. Before co-immunoprecipitation, we transfected the His-eEF2 plasmid into HEK293T cells. Full-length NFATc3 and RHR, but not the NHR or C-terminal domains, were specifically co-immunoprecipitated with His-eEF2 using Flag antibodies. Deletion of the NFATc3 RHR domain abolished the interaction between NFATc3 and eEF2 (Fig. 7F). The mammalian 2-hybrid assay revealed that RHR bound to eEF2 (Fig. S9F), indicating that NFATc3 binds to eEF2 directly. Moreover, deletion of the NFATc3 RHR domain abolished the NFATc3-induced downregulation of eEF2 phosphorylation level in MASMCs (Fig. 7G). PDGF-BB administration significantly increased His-eEF2 interaction with Flag-NFATc3 in MASMCs (Fig. S9G). Furthermore, eEF2 phosphorylation levels were lower in patients with TAAD (Fig. 7H). Similarly, we observed decreased eEF2 phosphorylation levels in aortas treated with BAPN/AngII and *Pcsk9*^DY^/AngII compared to levels in the control (Fig. 7I). eEF2 overexpression prevented VSMC contractile marker expression and OPN repression induced by TGF-*β*. Thus, eEF2 is associated with NFATc3 contractile-to-synthetic phenotype switching (Fig. S9H).

To elucidate the role of p-eEF2/eEF2 in NFATc3-mediated global protein synthesis and contractile-to-synthetic phenotypic switching, we treated MASMCs isolated from *Nfatc3*^fl/fl^ and *Nfatc3*^*smcko*^ aortas with A484954, a selective eEF2K inhibitor increasing eEF2 activity^26^. A484954 administration reversed the decreased global protein synthesis observed in VSMC–NFATc3 deficiency. In contrast, cabamiquine administration reversed the enhanced effects observed in *Nfatc3*^smcKI^ VSMCs, inhibiting eEF2 activity and increasing phosphorylation^27^ (Fig. 7J, Fig. S9I). Concomitantly, A484954 administration decreased VSMC contractile proteins induced by NFATc3 deficiency, whereas cabamiquine reversed the inhibitory effects in *Nfatc3*^smcKI^ VSMCs (Fig. S9J and S9K). Therefore, NFATc3 depletion impairs eEF2 binding to translational ribosomes, increases eEF2 phosphorylation, and suppresses translational elongation, resulting in lower protein synthesis and eventually inhibiting VSMC contractile-to-synthetic phenotype switching.

### 3.8 Cabamiquine supplementation reverses the effect of NFATc3 in AAD

**Figure 8:**
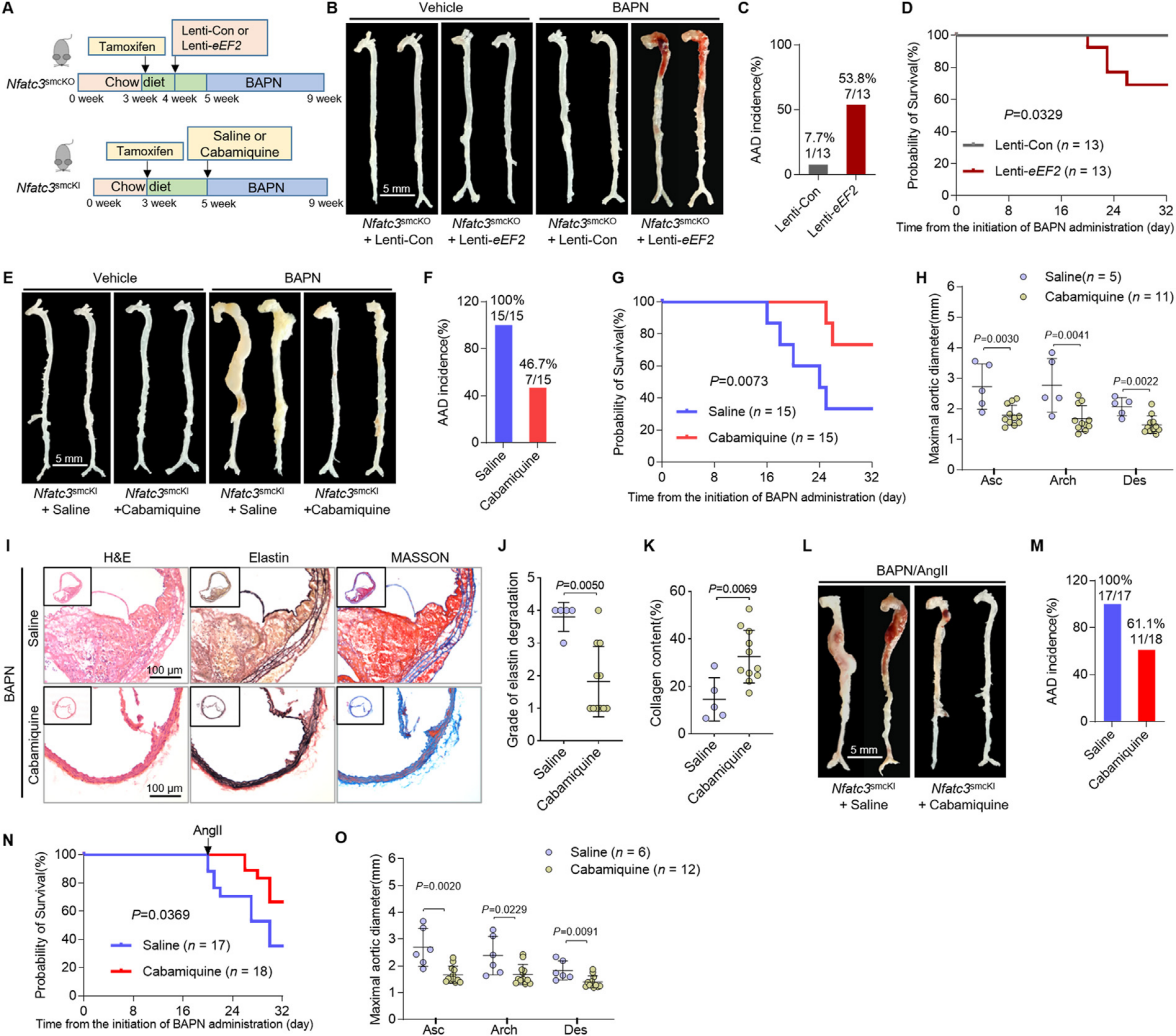
Supplementation with the eEF2 inhibitor cabamiquine mitigates the deleterious effects of NFATc3 overexpression on AAD development. (A) BAPN model establishment. (B–D) Four-week-old male *Nfatc3*^smcKO^ mice were treated with Lenti-Con or Lenti eEF2; 7 days after transfection, five-week-old mice were treated with vehicle or BAPN (0.6 g/kg/day) for 28 days (*n* = 13). (B) aorta macrographs. (C) AAD incidence. (D)The survival rate was estimated using Kaplan–Meier and compared using the log-rank test. (E–K) Five-week-old male *Nfatc3*^smcKI^ mice were treated with BAPN (0.6 g/kg/day) for 28 days. Saline and cabamiquine (3 mg/kg/day) were administered orally for 28 days (*n* = 15). (E) Aorta macrographs. (F) AAD incidence. (G) The survival rate was estimated using Kaplan–Meier and compared using the log-rank test. (H) Maximum aortic diameter. (I) H&E, van Geison (elastin), and Masson aorta staining. (J, K) Elastin degradation grade (J) and collagen content (K) in the aortic wall. (H–K) *n* = 5 for saline, *n* = 11 for cabamiquine. (L–O) Five-week-old *Nfatc3*^smcKI^ male mice were administered BAPN (0.3 g/kg/day) for 28 days before being infused with AngII (1000 ng/kg/min) for 3 days; saline and cabamiquine (3 mg/kg/day) were administered orally for 31 days (*n* = 17 for saline, *n* = 18 for cabamiquine). (L) Aorta macrographs. (M) AAD incidence. (N) The survival rate was estimated using Kaplan–Meier and compared using the log-rank test. (O) Maximum aortic diameter (saline: *n* = 6; cabamiquine: *n* = 12). Mice that died from aortic rupture were not included in the measurements. Data are presented as mean ± SD. (H, K, O) Unpaired Student’s *t*-test; two-tailed *P*-values. (J) Mann–Whitney *U*-test with the exact method; two-tailed *P*-values.

We used cabamiquine to further explore the therapeutic potential of NFATc3 in AAD by establishing the functional relationship between NFATc3 and eEF2 in an AAD model. More specifically, we constructed a BAPN-induced AAD model in *Nfatc3*^smcKO^ mice treated with Lenti-Con or Lenti-eEF2 and *Nfatc3*^smcKI^ mice treated with saline or cabamiquine (Fig. 8A). Higher eEF2 levels were observed in the MASMCs of mice treated with Lenti-eEF2 (Supporting Information Fig. S10A). Lenti-eEF2 administration increased BAPN-induced AAD progression in *Nfatc3*^smcKO^ mice (Fig. 8B–D, Fig. S10B). The aortic morphology of *Nfatc3*^smcKI^ mice treated with saline or cabamiquine was unchanged under vehicle conditions; nonetheless, cabamiquine inhibited BAPN-induced AAD progression in *Nfatc3*^smcKI^ mice treated with BAPN (Fig. 8E). The AAD mortality rate and incidence decreased in cabamiquine-treated *Nfatc3*^smcKI^ mice, and their aortic diameters were smaller than those of mice treated with saline (Fig. 8F–H). Cabamiquine decreased elastin and collagen degradation (Fig. 8I–K), without impacting blood pressure, body weight, or plasma lipids (Fig. S10C–S10F). Cabamiquine increased the p-eEF2/eEF2 ratio in the aortas of *Nfatc3*^smcKI^ mice treated with BAPN, consistent with our *in vitro* findings (Fig. S10G). Furthermore, cabamiquine inhibited the contractile-to-synthetic VSMC phenotypic switching induced by NFATc3 overexpression (Fig. S10H). Cabamiquine also inhibited AAD progression induced by NFATc3 overexpression in the BAPN/AngII and *Pcsk9*^DY^/AngII mouse models, including decreased AAD incidence, mortality rate, and aortic diameters. Higher p-eEF2 levels were observed in cabamiquine-treated *Nfatc3*^smcKI^ mice (Fig. 8L–O, Supporting Information Fig. S11A–S11F). Additionally, cabamiquine administration did not affect body weight, blood pressure, or plasma lipids in *Nfatc3*^smcKI^ mice (Fig. S11G–S11O). These findings indicate that cabamiquine inhibits AAD in an NFATc3-dependent manner and protects against aortic degeneration, dissection, and rupture.

## 4 Discussion

AAD is a lethal aortic disease with a high rupture risk. Therefore, investigating therapeutic strategies to prevent, inhibit, and reverse its progression is essential. Furthermore, VSMC homeostasis is crucial in AAD. Herein, we observed that VSMC–NFATc3 contributed to AAD progression, as supported by two mouse genotypes. Moreover, NFATc3 expression increased in the aortas of patients with aortic diseases (TAAD and AAA) and in mice with AAD. NFATc3 deficiency in VSMCs inhibited ECM degradation and the VSMC phenotypic switch, whereas NFATc3 overexpression had the opposite effect. Nuclear NFATc3 transcriptionally upregulated MMP9 and MMP2 levels and activity, aggravating ECM degradation. Moreover, cytoplasmic NFATc3 promoted protein synthesis by binding to eEF2 and inhibiting eEF2 phosphorylation in VSMC ribosomes, enabling VSMC contractile-to-synthetic phenotypic switching, ultimately exacerbating AAD development. Furthermore, cabamiquine, a novel antimalarial agent that inhibits protein synthesis by targeting eEF2, limited AAD progression. Collectively, our findings suggest the potential of VSMC–NFATc3 as a target for AAD treatment.

We used 3 AAD models to elucidate the effect of NFATc3 on AAD. BAPN is commonly used to induce aortic wall rupture and dilation by disrupting procollagen and tropoelastin cross-linking and destroying the aortic wall integrity^20^. BAPN combined with AngII induces a high incidence of aortic AAD rupture^28^. The AAV encoding *Pcsk9* D377Y (Asp374-to-Tyr mutant) targets hepatocytes by inducing hepatic low-density lipoprotein receptor degradation^15^, which was used to induce hyperlipidemia and combined with AngII infusion and a Western-type diet to induce AAA. VSMC–NFATc3 deficiency significantly decreased AAD development, whereas overexpression accelerated AAD progression in the specific VSMC-NFATc3-knockout and knock-in mice, respectively. Moreover, NFATc3 increased blood pressure in response to AngII stimulation. This contradicts prior findings related to VSMCs. Persisting [Ca^2+^]_i_ may be responsible for elevated blood pressure, activating the pro-hypertensive calcineurin/NFATc3 signaling cascade during AngII-induced hypertension^29^.

The NFAT family transcriptionally regulates gene expression in various physiological processes and pathological conditions^30^, ^31^, ^32^. NFATc3 functions in VSMC proliferation; however, the effect of NFATc3 isoforms on MMPs in AAD progression is unclear^33^. MMP activation contributes to arterial elastin degradation and promotes aortic ECM remodeling, with an essential role in AAD. The specific MMP cassette expression mechanism responsible for aortic ECM degradation could provide novel insights into isolated MMP inhibition^34^. MMP2 and MMP9 expression and activity increased in aortas with AAD, promoting AAD progression. Meanwhile, their inhibition attenuated AAD formation, revealing a promising strategy to mitigate AAD development^35^^,^^36^. MMP9 and MMP2 expression was significantly decreased in VSMC-NFATc3-deficient mouse aortas following BAPN administration. Nuclear NFATc3 transcriptionally upregulated MMP9 and MMP2 levels. Therefore, NFATc3-mediated ECM degradation in VSMCs requires MMP9 and MMP2 overexpression. Transcription factors may modulate histone modifications to regulate gene expression. NFATc3, for example, may recruit co-activators with histone acetyltransferase (HAT) and histone methyltransferase (HMT) activities, potentially leading to the enrichment of H3K27ac and H3K4me3 on the *Mmp9* and *Mmp2* promoters. These histone modifications are generally associated with active chromatin states, which could promote transcriptional activation^37^^,^^38^. By possibly recruiting co-activators such as CBP/p300 for H3K27 acetylation and the COMPASS complex for H3K4 trimethylation, NFATc3 may enhance transcriptional activity at these promoters, potentially facilitating the expression of MMP9 and MMP2^39^. This activity may promote ECM degradation and contribute to the pathological remodeling observed in AAD. Therefore, NFATc3 may not just function as a DNA-binding factor but also as a chromatin remodeler, potentially influencing gene expression through epigenetic modifications. Further research is needed to explore the specific interactions between NFATc3 and chromatin modifiers and to understand their roles in different pathological conditions.

NFATc3 comprises the NHR, RHR containing the DNA-binding motif, and carboxy(C)-terminal domains^40^. Calcineurin, a calcium/calmodulin-dependent phosphatase, regulates NFATc3 protein activity. Calcineurin interacts with the NHR domain to regulate NFATc3 translocation from the cytoplasm to the nucleus in activated cells^40^. RHR confers DNA-binding specificity and shares similar structural homology with REL proteins, which can bind to regulatory elements in genes^40^. Cytoplasmic NFATc3 expression increased during AAD, suggesting its essential role in VSMCs. Moreover, transcription factors participate in non-transcriptional regulatory processes^41^^,^^42^. NFAT plays a non-transcriptional role in platelets, as its inhibition enhances activated platelet aggregation and exacerbates Gram-negative bacterial septicemia^23^.

Increased global protein synthesis is related to cell proliferation and migration in various cell types^43^, ^44^, ^45^. VSMCs can switch from a contractile to a pro-synthetic phenotype, characterized by reduced contractile protein expression and increased proliferation, migration, and secretion. The puromycin incorporation assay is a non-radioactive method widely used to label nascent proteins and assess the protein synthesis rate^46^. Increased global protein synthesis contributes to cell proliferation and migration. Conversely, reduced protein biosynthesis is associated with inhibited VSMC proliferation^47^. However, the relationship between protein synthesis and phenotypic transition in VSMCs has not been thoroughly investigated. Here, we revealed that NFATc3 exerts a non-genomic function while precisely localizing in the VSMC ribosome, which regulates protein synthesis and translational elongation. Furthermore, VSMCs undergo a phenotypic switch during AAD development^48^. Moreover, increased protein synthesis promotes VSMC phenotypic switching.

The role of NFAT in platelets reveals its non-genomic functions, particularly in the regulation of platelet activation, aggregation, neutrophil interactions, NETosis, and coagulation^23^. These findings offer valuable insights that inspire further investigation into the non-genomic roles of NFATc3 in VSMCs, potentially identifying additional regulatory mechanisms. Given the critical role of NFATc3 in VSMC phenotype differentiation through its involvement in protein synthesis, our findings suggest a complex underlying molecular mechanism. We identified several NFATc3-interacting proteins, including 11 40S ribosomal proteins, 19 60S ribosomal proteins, 5 elongation factors, and 1 initiation factor, which are significantly enriched during translation. The direct localization of NFATc3 in the ribosome indicates that it may play a crucial role in the elongation process. Protein synthesis can be divided into initiation, elongation, termination, and ribosome recycling, each requiring a specific translation factor^49^. eEF2 is a member of the GTP-binding elongation factor family and catalyzes peptidyl-tRNA translocation from the ribosomal A site to the P site during protein synthesis; it is highly expressed in tumor cells with fast protein synthesis^50^. Therefore, targeting eEF2 is an effective strategy for slowing tumor growth. eEF2 activity is regulated by eEF2K, which phosphorylates eEF2 at Thr56 (p-eEF2) and inhibits elongation, whereas eEF2 can be dephosphorylated by PP2A, thereby promoting protein synthesis, phosphorylated eEF2 is inactivated and removed from the ribosome^49^^,^^51^^,^^52^. Enhanced eEF2 expression in VSMCs has been reported in spontaneously hypertensive rats^53^, and increased eEF2 and decreased p-eEF2 levels were consistently observed in aortas with AAD. Notably, we revealed a novel function of NFATc3 in regulating protein synthesis by interacting with eEF2 at Thr56 and inhibiting its phosphorylation in ribosomes, without affecting eEF2K or PP2A. Furthermore, activating eEF2 with A-484954 reversed the NFATc3 deficiency-mediated attenuation of global protein synthesis and the VSMC phenotypic switch. More importantly, eEF2 upregulation reversed the NFATc3 deficiency-mediated protective effect in AAD progression. Contrastingly, cabamiquine eEF2 inhibition reversed the enhanced global protein synthesis and the VSMC phenotypic switch mediated by NFATc3 overexpression. NFATc3 binding to eEF2 *via* RHR, the DNA-binding domain, decreased eEF2 phosphorylation, thereby increasing its protein synthesis-promoting activity. However, further investigation is needed to determine the specific binding sites. These findings suggest that NFATc3-mediated regulation is critical for the VSMC phenotypic switch and protein synthesis.

Cabamiquine, an antimalarial agent that antagonizes the *Plasmodium* parasite by targeting eEF2 and inhibiting protein synthesis, is in phase I clinical trials to treat *Plasmodium falciparum* sporozoites (https://clinicaltrials.gov; NCT04250363, NCT03261401) due to its effectiveness and safety^27^. In this study, cabamiquine supplementation significantly attenuated the VSMC phenotypic switch and inhibited AAD development in the BAPN, BAPN/AngII, and *Pcsk9*^DY^/AngII-induced AAD models in an NFATc3-dependent manner.

Our *in vivo* findings provide new insights into the biological role of cabamiquine in pharmacological eEF2 inhibition for AAD prevention and treatment. Notably, compared to the widely used captopril, which acts as an ACE inhibitor and primarily reduces blood pressure and vascular stress^54^, cabamiquine directly impacts the cellular mechanisms involved in AAD progression. While captopril offers hemodynamic benefits that slow aneurysm expansion by lowering blood pressure, the ability of cabamiquine to inhibit VSMC phenotypic switching *via* suppressing eEF2 activity offers a more direct approach for disrupting the cellular pathways underlying AAD progression.

Overall, cabamiquine may offer a complementary therapeutic benefit in AAD by addressing both molecular pathology and hemodynamic factors, making it a promising addition to current clinical therapies.

## 5 Conclusions

We revealed the effect of VSMC–NFATc3 on AAD development. NFATc3 expression was elevated, followed by ECM degradation and VSMC phenotypic transition enhancement from the contractile to synthetic phenotype in AAD. Nuclear NFATc3 specifically targeted and transcriptionally upregulated MMP9 and MMP2 levels and promoted ECM degradation. Cytoplasmic NFATc3 inhibited eEF2 phosphorylation in VSMCs, increasing protein synthesis, downregulating differentiation markers, and ultimately exacerbating AAD development. Hence, NFATc3 may be a novel potential therapeutic target for AAD, and cabamiquine may be used as a new therapeutic agent.

## Author contributions

Xiu Liu: Writing – review & editing, Writing – original draft, Resources, Investigation, Funding acquisition, Data curation, Conceptualization. Li Zhao: Writing – original draft, Validation, Supervision, Investigation, Conceptualization. Deshen Liu: Writing – original draft, Visualization, Resources, Data curation, Conceptualization. Lingna Zhao: Writing – original draft, Resources, Investigation, Conceptualization. Yonghua Tuo: Resources, Formal analysis, Conceptualization. Qinbao Peng: Visualization, Software, Investigation. Fangze Huang: Visualization, Software, Conceptualization. Zhengkun Song: Visualization, Software, Conceptualization. Chuanjie Niu: Visualization, Investigation. Xiaoxia He: Validation, Investigation. Yu Xu: Visualization, Investigation. Jun Wan: Visualization, Investigation. Peng Zhu: Investigation. Zhengyang Jian: Investigation. Jiawei Guo: Investigation. Yingying Liu: Investigation. Jun Lu: Writing – original draft, Visualization, Investigation, Conceptualization. Sijia Liang: Visualization, Resources, Investigation. Shaoyi Zheng: Writing – review & editing, Writing – original draft, Visualization, Validation, Resources, Investigation, Funding acquisition, Formal analysis, Conceptualization.

## Conflicts of interest

The authors declare no competing interests.

## Appendix A Supporting information

The following is the Supporting information to this article:
