# Supplementary material for: Cytoplasmic and nuclear NFATc3 cooperatively contributes to vascular smooth muscle cell dysfunction and drives aortic aneurysm and dissection
Source: Acta Pharm Sin B. 2025 May 21;15(7):3663–84. doi: 10.1016/j.apsb.2025.05.016 (PMC12278440; doi:10.1016/j.apsb.2025.05.016)
Supplement: Multimedia component 1 [file mmc1.pdf]

Supporting Information for

Original article

Cytoplasmic and nuclear NFATc3 cooperatively contributes to vascular smooth muscle cell dysfunction and drives aortic aneurysm and dissection

Xiu Liu[a,*,†], Li Zhao[a,†], Deshen Liu[a,†], Lingna Zhao[a,†], Yonghua Tuo[b], Qinbao Peng[a], Fangze Huang[a], Zhengkun Song[a], Chuanjie Niu[a], Xiaoxia He[a], Yu Xu[a], Jun Wan[a], Peng Zhu[a], Zhengyang Jian[c], Jiawei Guo[d], Yingying Liu[e], Jun Lu[a], Sijia Liang[f,*], Shaoyi Zheng[a,*]

[a]*Department of Cardiovascular Surgery, Nanfang Hospital, Southern Medical University, Guangzhou 510515, China*

[b]*Department of Neurosurgery, the Second Affiliated Hospital of Guangzhou Medical University, Guangzhou 510260, China*

[c]*Center for Drug Inspection of Guizhou Medical Products Administration, Guiyang 550081, China*

[d]*Department of Pharmacology, School of Medicine, Yangtze University, Jingzhou 434023, China*

[e]*Guangzhou Women and Children's Medical Center, Guangdong Provincial Clinical Research Center for Child Health, Guangzhou 510623, China*

[f]*Department of Pharmacology, Zhongshan School of Medicine, Sun Yat-sen University, Guangzhou 510080, China*

Received 4 June 2024; received in revised form 13 October 2024; accepted 20 December 2024

[*]Corresponding authors.

E-mail addresses: liux266@smu.edu.cn (Xiu Liu), zhsy@smu.edu.cn (Shaoyi Zheng), liangsj@mail.sysu.edu.cn (Sijia Liang).

[†]These authors made equal contributions to this work.

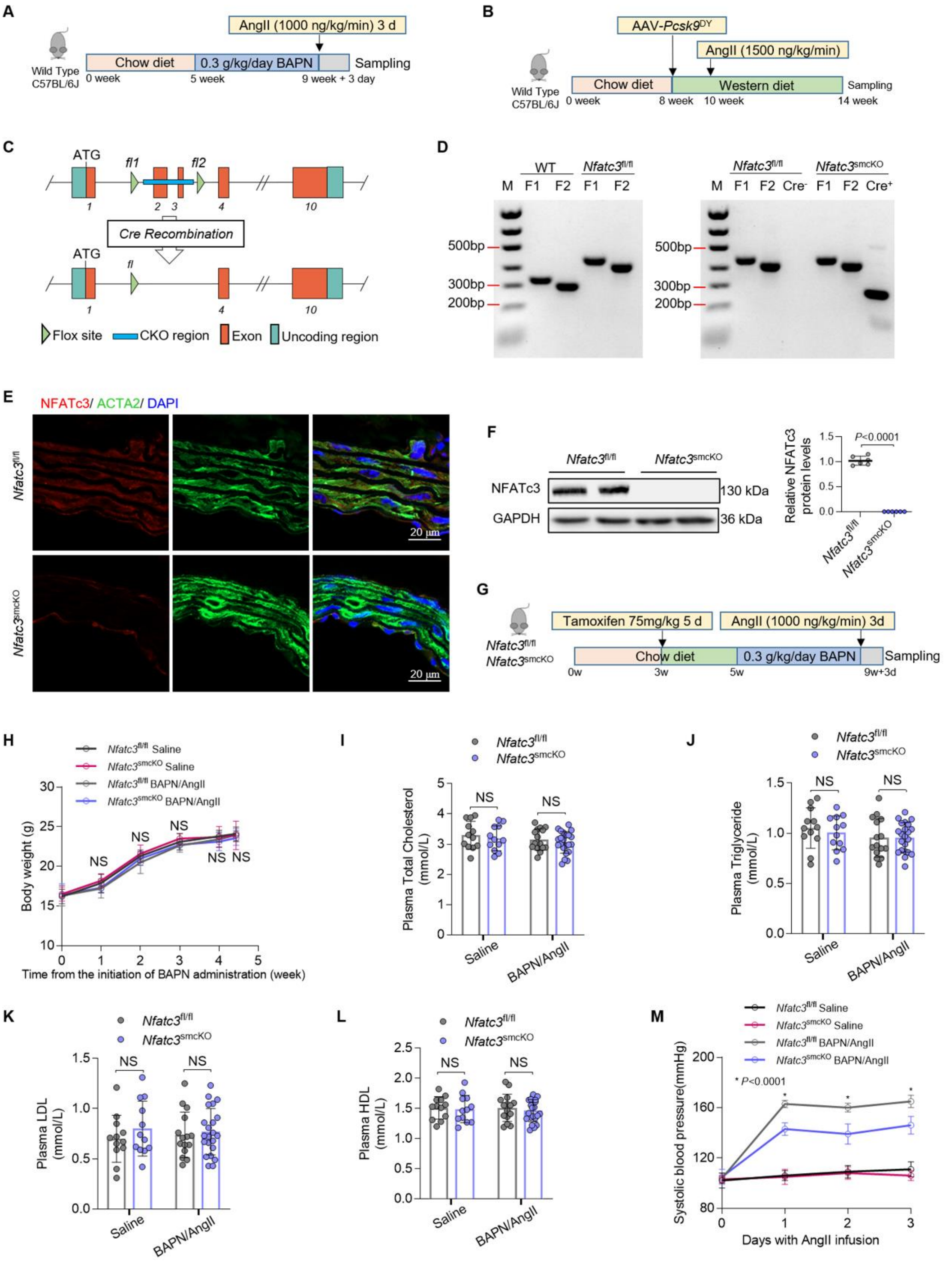

Figure S1 VSMC-NFATc3 deficiency inhibited AAD progression in the BAPN/AngII model. (A, B) BAPN/angiotensin II (AngII) (A) and *Pcsk9*^{DY}/AngII (B) models in C57BL/6J mice. (C) Strategy for generation of *Nfatc3*$^{fl/fl}$ mice. (D) Mice were PCR-genotyped using specific primers for fl1 (F1), fl2 (F2), or *Myh11*-Cre. Mice harboring fl1, fl2, and *Mhy11*-Cre were considered *Nfatc3*^{smcKO}; those harboring fl1 and fl2, but not *Mhy11*-Cre, were considered *Nfatc3*$^{fl/fl}$. (E) Representative images of NFATc3 and

ACTA2 expression using immunofluorescence staining in the aortas of *Nfatc3*$^{fl/fl}$ and *Nfatc3*^{smcKO} mice (n = 6). (F) NFATc3 protein levels in VSMCs isolated from *Nfatc3*$^{fl/fl}$ and *Nfatc3*^{smcKO} mice (n = 6). (G–M) Five-week-old *Nfatc3*$^{fl/fl}$ or *Nfatc3*^{smcKO} male mice were treated with BAPN (0.3 g/kg/day) for 28 days and infused with angiotensin II (1000 ng/kg/min) for 3 days (n = 22; n = 12 for *Nfatc3*$^{fl/fl}$ or *Nfatc3*^{smcKO} with saline). (G) Schematic of BAPN/AngII model establishment in *Nfatc3*$^{fl/fl}$ and *Nfatc3*^{smcKO} mice. (H–M) Body weight (H), serum levels of TC (I), TG (J), LDL (K), HDL (L), and systolic blood pressure (M) in *Nfatc3*$^{fl/fl}$ and *Nfatc3*^{smcKO} mice with *β*-aminopropionitrile (BAPN)/AngII (n = 15 for *Nfatc3*$^{fl/fl}$ and n = 22 for *Nfatc3*^{smcKO}; mice that died of aortic rupture were not included in measurements). Data are presented as mean ± SD. (F) Non-normally distributed data were analyzed using the Mann–Whitney U-test with exact method. (H–M) Two-way ANOVA with Tukey's correction; adjusted *P*-values are shown. NS, no significance ($P > 0.05$).

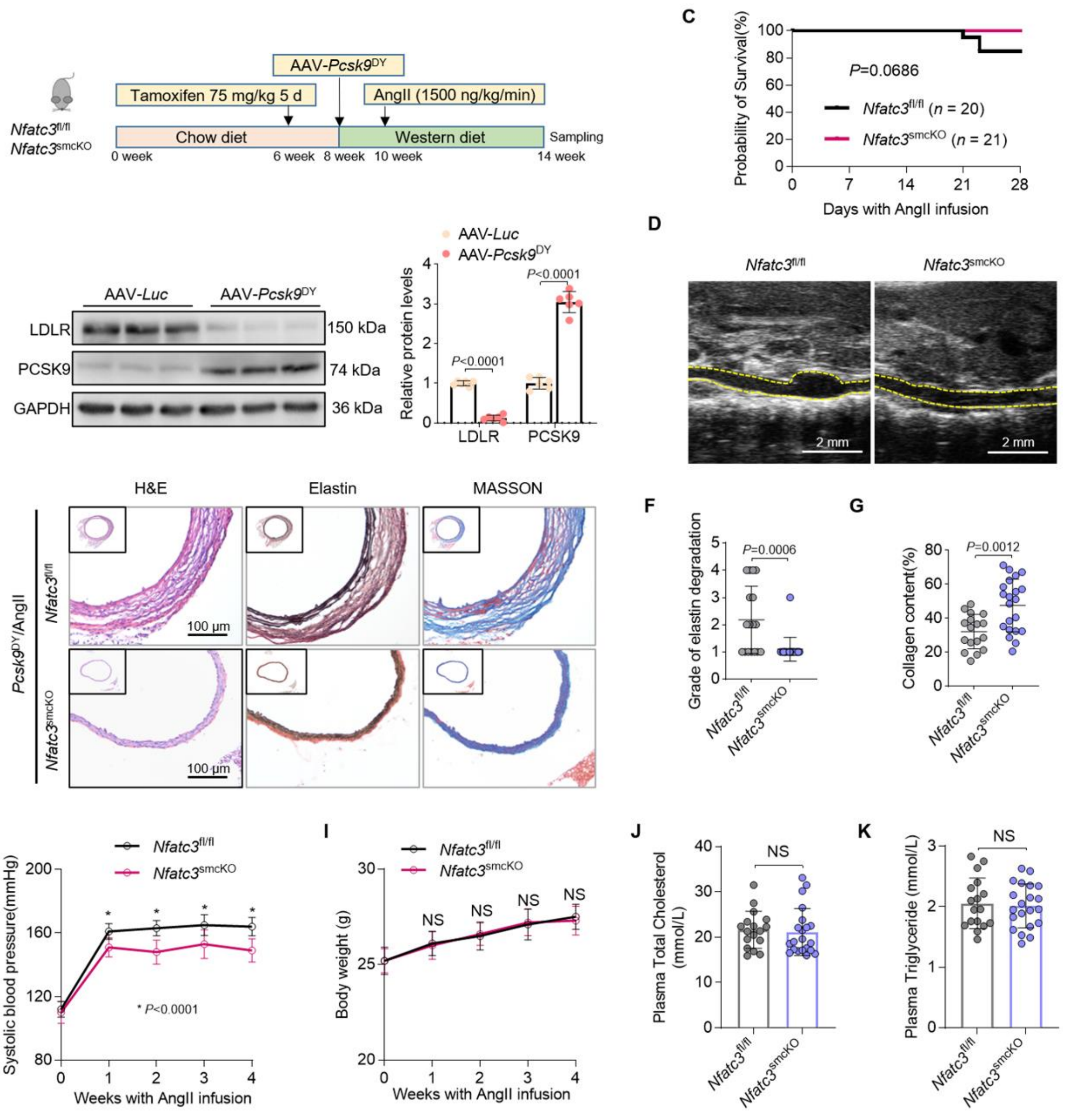

Figure S2 VSMC-NFATc3 deficiency inhibited AAA progression in the *Pcsk9*^{DY}/AngII model. (A–H) Eight-week-old *Nfatc3*$^{fl/fl}$ or *Nfatc3*^{smcKO} mice were injected intraperitoneally with AAV-*Pcsk9*^{DY} and fed a Western-type diet. After 2 weeks, they were infused with AngII (1500 ng/kg/min) for another 4 weeks (n = 20 for *Nfatc3*$^{fl/fl}$ and n = 21 for *Nfatc3*^{smcKO}). (A) Schematic of AAV-*Pcsk9*^{DY}/AngII model establishment in *Nfatc3*$^{fl/fl}$ and *Nfatc3*^{smcKO} mice. (B) Eight-week-old wild-type mice were intraperitoneally injected with AAV-*Luc or* AAV-*Pcsk9*^{DY} and fed a Western-type diet for 6 weeks. The panel shows hepatic low-density lipoprotein receptor (LDLR) and PCSK9 expression in the mice (n = 6). (C) Survival curves were analyzed using the Kaplan–Meier method and compared using log-rank tests. (D) Representative ultrasound images of abdominal aorta. (E) Representative hematoxylin and eosin, van Geison (elastin), and Masson staining of mouse abdominal aorta. (F, G) Grade of elastin degradation (F) and collagen content (G) in aortic wall (n = 17 for *Nfatc3*$^{fl/fl}$ and n = 21

for *Nfatc3*^{smcKO}). (H–K) Systolic blood pressure (H), body weight (I), and serum TC (J) and TG (K) levels in *Nfatc3*$^{fl/fl}$ and *Nfatc3*^{smcKO} mice (n = 17 for *Nfatc3*$^{fl/fl}$ and n = 21 for *Nfatc3*^{smcKO}, mice that died of aortic rupture were not included in the measurements). The assessment was performed after an AAV-*Pcsk9*^{DY}/AngII administration. Data are presented as mean ± SD. Log-rank tests were used for (C). (B, F–K) The normality test failed, and the Mann–Whitney U-test with the exact method was used. For all other data, the normality test passed, and the unpaired Student's t-test was used. Two-tailed P-values are shown. NS, no significance ($P > 0.05$).

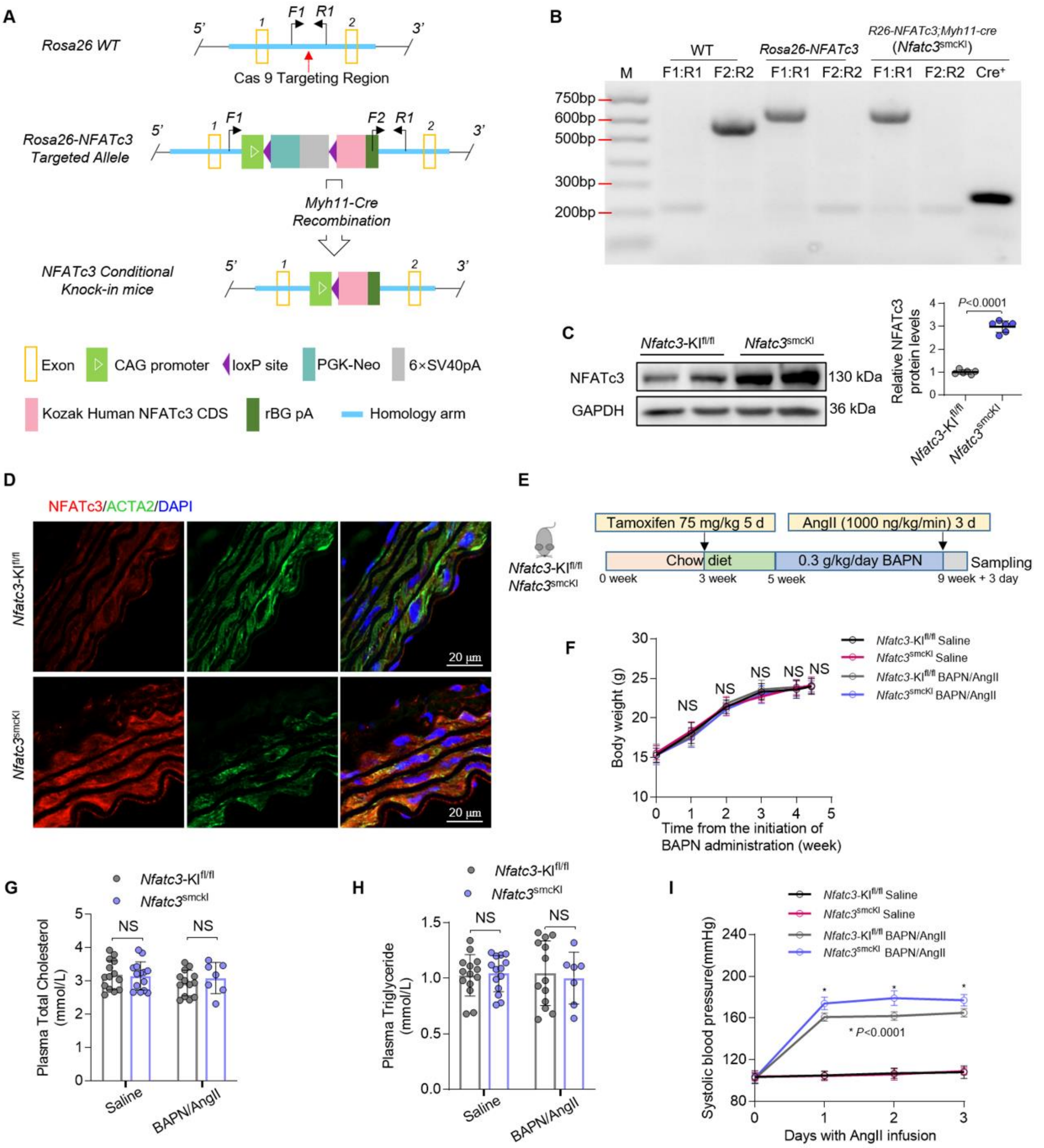

Figure S3 VSMC-NFATc3 overexpression aggravates AAD progression in the BAPN/AngII model. (A) Generation of *Nfatc3*^{smcKI} mice. (B) Identification of *Nfatc3*-KI$^{fl/fl}$ and *Nfatc3*^{smcKI} mice. (C) NFATc3 protein levels in the VSMCs isolated from *Nfatc3*-KI$^{fl/fl}$ and *Nfatc3*^{smcKI} mice (n = 6). (D) Representative images of NFATc3 and ACTA2 expression using immunofluorescence staining in the aortas of each group (n = 6). (E) Schematic of BAPN/AngII model establishment in *Nfatc3*-KI$^{fl/fl}$ and *Nfatc3*^{smcKI} mice (n = 19 for *Nfatc3*-KI$^{fl/fl}$; n = 20 for *Nfatc3*^{smcKI}; n = 14 for *Nfatc3*-KI$^{fl/fl}$ or *Nfatc3*^{smcKI} with saline). (F–I) Body weight (G), TC (G) and TG (H) serum levels, and systolic blood pressure (I) in *Nfatc3*$^{fl/fl}$ and *Nfatc3*^{smcKO} mice with BAPN/AngII (n = 13 for *Nfatc3*-KI$^{fl/fl}$ and n = 7 for *Nfatc3*^{smcKI}; mice that died of aortic rupture were not included in the measurements). Data are presented as mean ± SD. (C)

Unpaired Student's *t*-test was used. Two-tailed *P*-values are shown. (F–I) Two-way ANOVA with Tukey's correction was used; the adjusted *P*-values are shown. NS, no significance ($P > 0.05$).

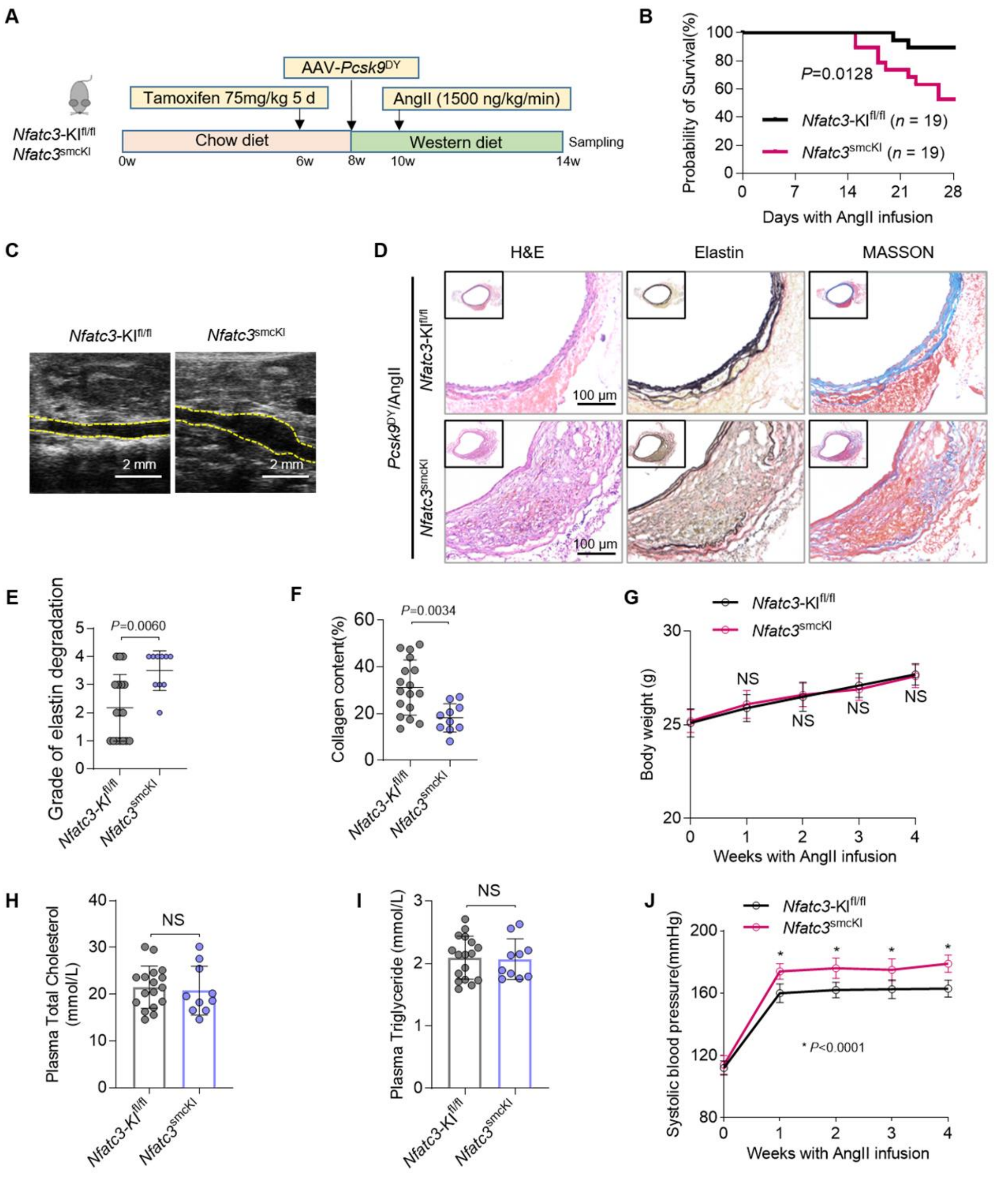

Figure S4 VSMC-NFATc3 overexpression aggravates AAA progression in the *Pcsk9*^{DY}/AngII model. (A–J) Eight-week-old *Nfatc3*-KI$^{fl/fl}$ and *Nfatc3*^{smcKI} mice were injected with AAV-*Pcsk9*^{DY} intraperitoneally and fed a Western-type diet. After 2 weeks, they were infused with AngII (1500 ng/kg/min) for another 4 weeks (n = 19). (A)

Schematic of AAV-*Pcsk9*^{DY}/AngII model establishment in *Nfatc3*-KI$^{\text{fl/fl}}$ and *Nfatc3*^{smcKI} mice. (B) Survival curves were analyzed using the Kaplan–Meier method and compared using log-rank tests. (C) Representative ultrasound images of the abdominal aorta. (D) Representative hematoxylin and eosin, van Geison (elastin), and Masson trichrome staining of mouse abdominal aorta. (E, F) Grade of elastin degradation (E) and collagen content (F) in the aortic wall. (G–J) Body weight (E), TC (H) and TG (I) serum levels, and systolic blood pressure (J) in *Nfatc3*-KI$^{\text{fl/fl}}$ and *Nfatc3*^{smcKI} mice (n = 17 for *Nfatc3*$^{\text{fl/fl}}$ and n = 10 for *Nfatc3*^{smcKO}; mice that died of aortic rupture were not included in the measurements). Data are presented as mean ± SD. Log-rank tests were used for (B). (E–J) The normality test failed, and the Mann–Whitney U-test with exact method was used. For all other data, the normality test passed, and unpaired Student's t-test was used. Two-tailed P-values are shown. NS, no significance ($P > 0.05$).

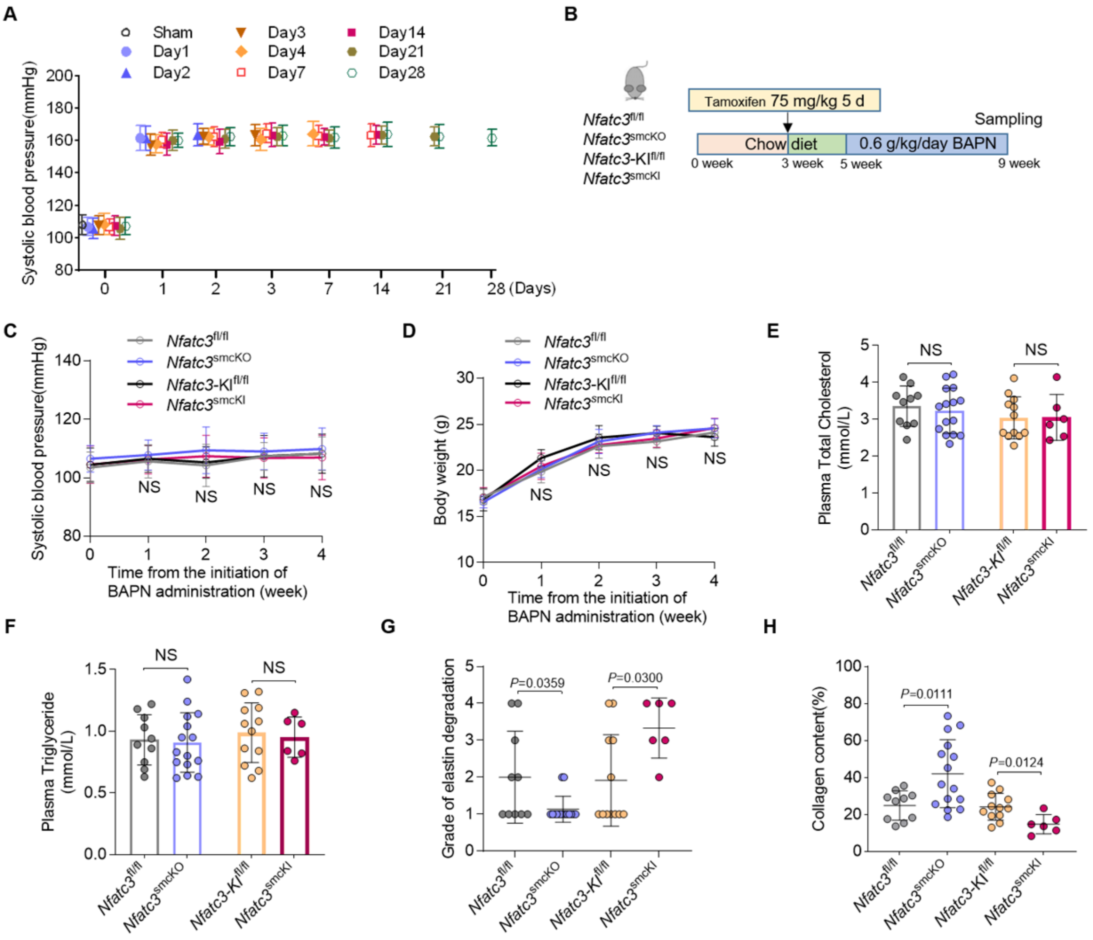

Figure S5 VSMC-NFATc3 facilitates AAD progression in the BAPN model. (A) Systolic blood pressure of mice at different days of AngII administration, as indicated. (B–H) Five-week-old male *Nfatc3*$^{\text{fl/fl}}$ (n = 15), *Nfatc3*^{smcKO} (n = 15), *Nfatc3*-KI$^{\text{fl/fl}}$ (n =

17), and *Nfatc3*^{smcKI} (n = 18) mice were treated with BAPN (0.6 g/kg/day) for 28 days. (B) Schematic of establishment of the BAPN model. (C-F) Systolic blood pressure (C), body weight (D), and TC (E) and TG (F) serum levels in mice as indicated. (G–H), Grade of elastin degradation (G) and collagen content (H) in the aortic wall (n = 10 for *Nfatc3*$^{fl/fl}$, n = 15 for *Nfatc3*^{smcKO}, n = 12 *Nfatc3*-KI$^{fl/fl}$, and n = 6 *Nfatc3*^{smcKI}; mice that died of aortic rupture were not included in the measurements). Data are presented as mean ± SD. (C–H) Two-way ANOVA with Tukey's correction was used; the adjusted P-values are shown. NS, no significance (P > 0.05).

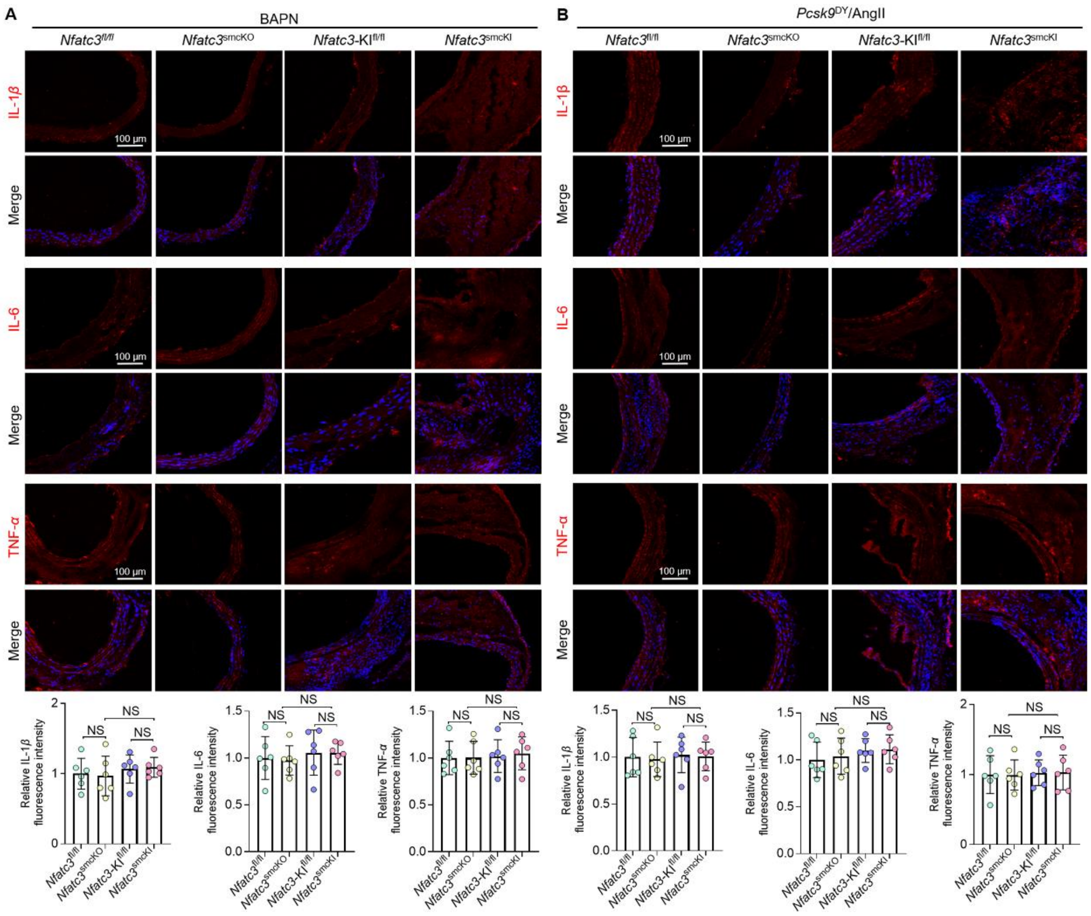

Figure S6 VSMC-NFATc3 does not affect the expression of inflammatory factors in the aortic wall of AAD mice. (A) Representative immunofluorescence images of IL-1*β*, IL-6, and TNF-*α* in ascending aortas of *Nfatc3*$^{fl/fl}$, *Nfatc3*^{smcKO}, *Nfatc3*-KI$^{fl/fl}$ and *Nfatc3*^{smcKI} mice treated with vehicle or BAPN for 28 days (n = 6)， (B) Eight-week-old *Nfatc3*$^{fl/fl}$, *Nfatc3*^{smcKO}, *Nfatc3*-KI$^{fl/fl}$ and *Nfatc3*^{smcKI} mice were intraperitoneally injected with AAV-*Pcsk9*^{DY} and fed a Western-type diet. After 2 weeks, they were infused with AngII (1500 ng/kg/min) for another 4 weeks. The representative immunofluorescence images of IL-1*β*, IL-6, and TNF-*α* in ascending aortas (n = 6) are

shown**.** Data are presented as mean ± SD. (A, B) As the normality test failed, the Mann–Whitney U-test with exact method was used. For other data that passed the normality test, unpaired Student's *t*-test was used. Two-tailed *P*-values are shown. NS, no significance ($P > 0.05$).

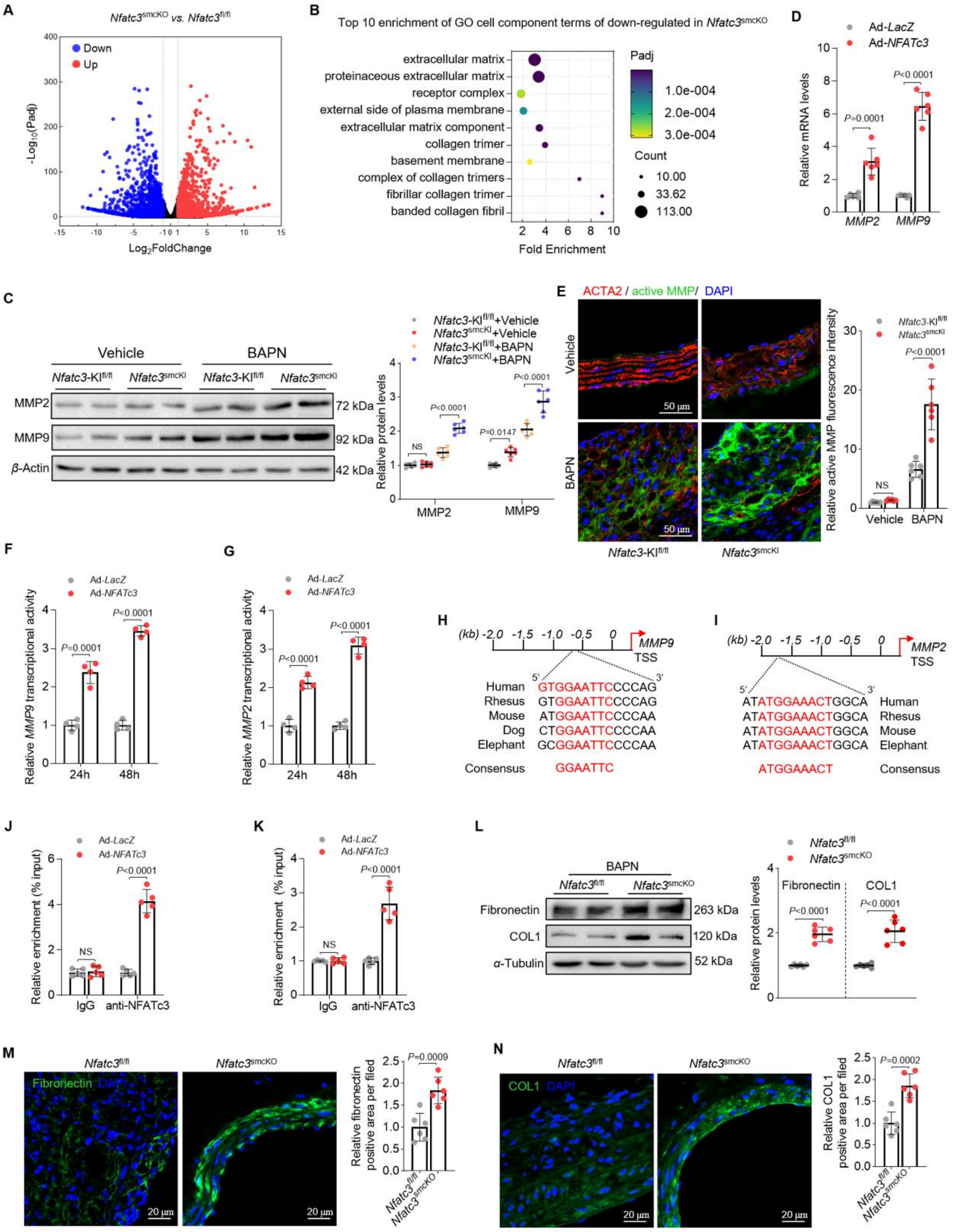

Figure S7 NFATc3 promotes ECM degradation by transcriptionally regulating MMP9 and MMP2 expression in VSMCs. (A) Volcano plots of the differentially expressed

genes in the aortas of *Nfatc3*^{smcKO} mice compared to *Nfatc3*$^{fl/fl}$ mice treated with BAPN (0.6 g/kg/day) for 28 days (*n* = 3). (B) Top 10 ranking biological processes contributing to NFATc3 function. (C) Western blots of MMP2 and MMP9 in the aortas of *Nfatc3*-KI$^{fl/fl}$ and *Nfatc3*^{smcKI} mice treated with vehicle or BAPN for 28 days (*n* = 6). (D) *MMP2* and *MMP9* mRNA levels in HASMCs infected with Ad-LacZ and Ad-NFATc3 (*n* = 6). (E) Representative immunofluorescence images of *in situ* zymography and immunostaining for ACTA2 in ascending aortas of *NFATc3*-KI$^{fl/fl}$ and *NFATc3*^{smcKI} mice treated with vehicle or BAPN for 28 days (*n* = 6). (F, G) Nuclear run-on assay for assessment of nascent *MMP9* and *MMP2* mRNA levels in HASMCs for 24 or 48 h after transfection with Ad-LacZ and Ad-*NFATc3* (*n* = 4). (H, I) Schematic of the binding site of NFATc3 on the *MMP9* promoter (H) and *MMP2* (I) gene and evolutionary conservation of the sequence recognized by NFATc3. (J, K) ChIP-qPCR analysis of NFATc3 enrichment on the *MMP9* (J) and *MMP2* (K) promoters in HASMCs (*n* = 5). (L–N), Five-week-old male *Nfatc3*$^{fl/fl}$ and *Nfatc3*^{smcKO} mice were treated with BAPN (0.6 g/kg/day) for 28 days. (L), Representative Western blots and quantification of fibronectin and COL1 in mouse aortic tissues. (M, N) Representative immunofluorescence images of fibronectin (M) and COL1 (N) in mouse aortic tissues. n = 6. Data are presented as mean ± SD. (C–G, J, K) Two-way ANOVA with Tukey's correction was used; adjusted *P*-values are shown. (L–N) Unpaired Student's *t*-test was used. Two-tailed *P*-values are shown. NS, no significance ($P > 0.05$).

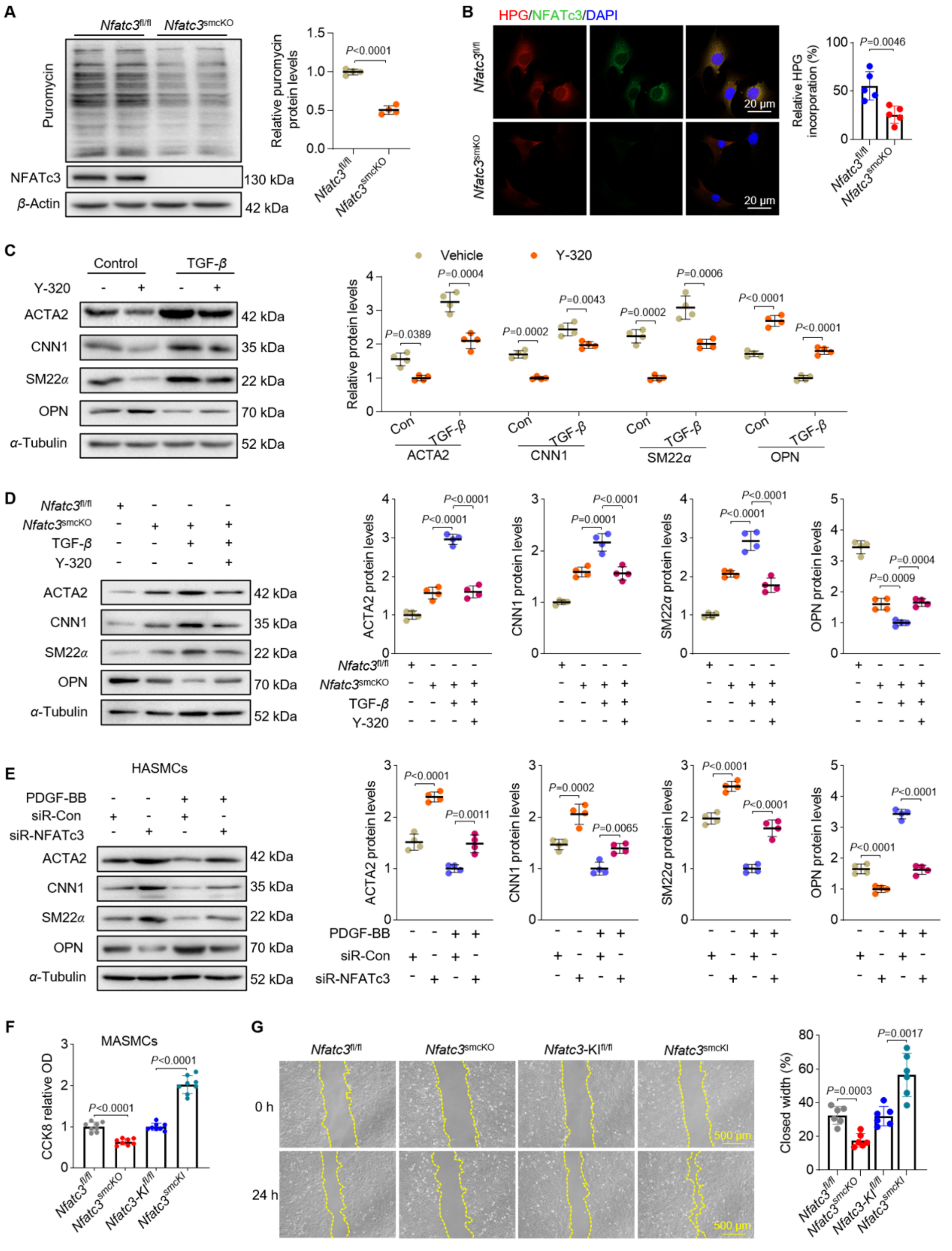

Figure S8 Cytoplasmic NFATc3 facilitates VSMC contractile-to-synthetic phenotype switching by promoting global protein synthesis. (A) Representative Western blots and quantification of puromycin incorporation assays from MASMCs isolated from *Nfatc3*$^{fl/fl}$ and *Nfatc3*^{smcKO} mice (n = 4). (B) Representative images and quantification of HPG incorporation assay in VSMCs isolated from the aortas of *Nfatc3*$^{fl/fl}$ and *Nfatc3*^{smcKO} mice (n = 5). (C) Representative Western blots and quantification of ACTA2, CNN1, SM22*α*, and OPN in MASMCs treated with TGF-*β* (10 μg/L) for 48 h

before a 24-h Y-320 (2 μmol/L) treatment (*n* = 4). (D) Representative Western blots and quantification of ACTA2, CNN1, SM22*α*, and OPN in MASMCs isolated from the aortas of *Nfatc3*$^{fl/fl}$ and *Nfatc3*^{smcKO} mice and treated with TGF-*β* (10 μg/L) for 48 h before a 24-h Y-320 (2 μmol/L) treatment (*n* = 4). (E) Representative Western blots and quantification of ACTA2, CNN1, SM22*α*, and OPN in HASMCs infected with siR-Con, and siR-NFATc3 for 48 h before 24-h PDGF-BB treatment (*n* = 4). (F), Relative OD values of CCK8 in *Nfatc3*$^{fl/fl}$, *Nfatc3*^{smcKO}, *Nfatc3*-KI$^{fl/fl}$, and *Nfatc3*^{smcKI} VSMCs (*n*=8). (G), Representative images of wound-healing assays and relative migration rates of VSMCs isolated from *Nfatc3*$^{fl/fl}$, *Nfatc3*^{smcKO}, *Nfatc3*-KI$^{fl/fl}$, and *Nfatc3*^{smcKI} mice (*n*=6). Data are presented as mean ± SD. (A) Unpaired Student's *t*-test was used. Two-tailed *P*-values are shown. (C) Two-way ANOVA with Tukey's correction was used; the adjusted *P*-values are shown. (D–G) One-way ANOVA followed by Tukey's correction was used; the adjusted *P*-values are shown.

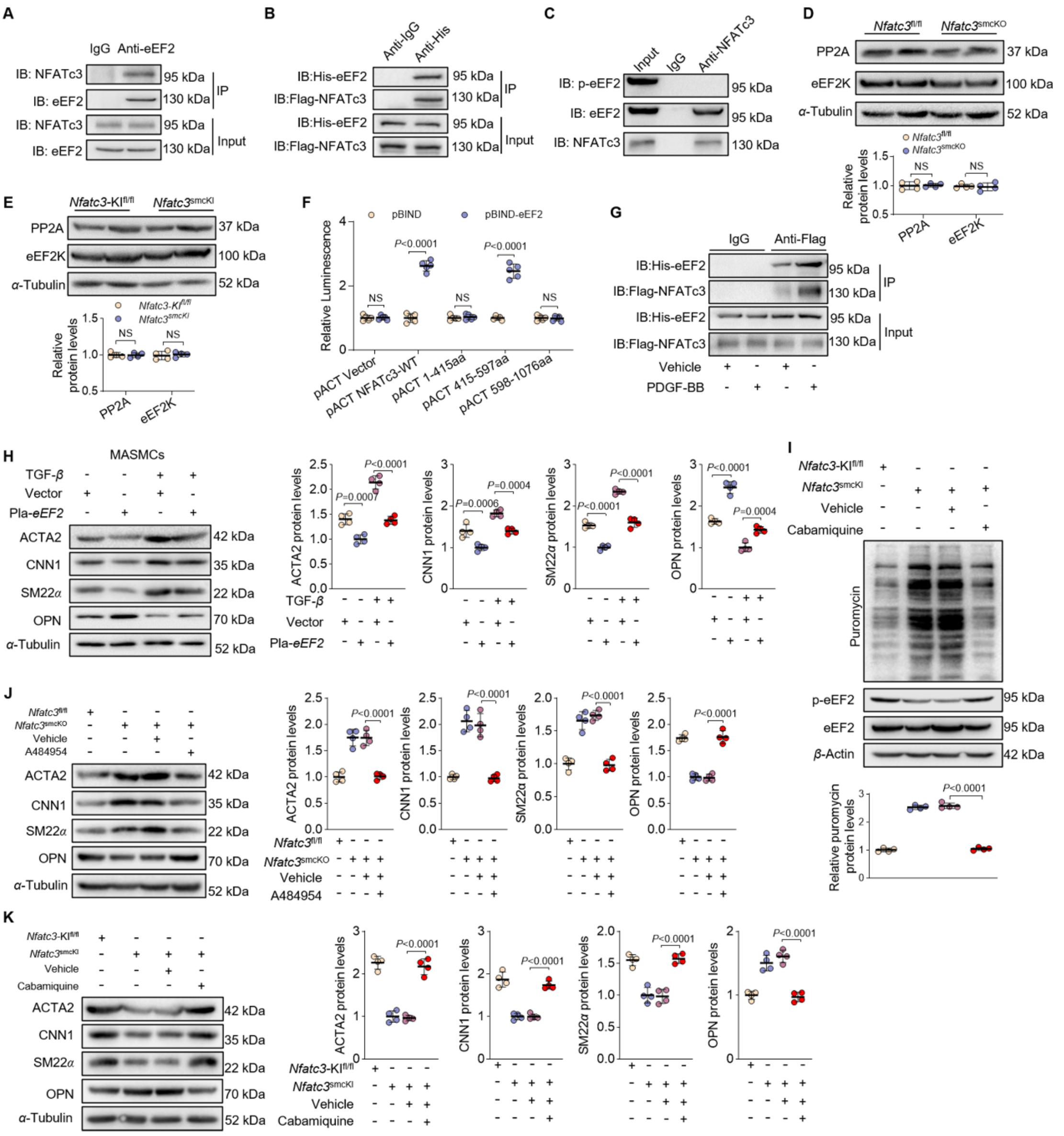

Figure S9 NFATc3 facilitates global protein synthesis by suppressing eEF2 phosphorylation. (A) Co-immunoprecipitation of MASMC lysates with anti-eEF2 antibody. NFATc3 protein levels were determined *via* Western blot. (B) HEK293T cells were co-transfected with the His-*eEF2* and Flag-*NFATc3* plasmids. Cell lysates were immunoprecipitated with anti-His antibody, and precipitates were analyzed using immunoblotting with anti-Flag antibody. (C) Co-immunoprecipitation of MASMC lysates with anti-NFATc3 antibodies; the precipitates were analyzed using immunoblotting with anti-eEF2 and anti-p-eEF2 antibodies. (D) Western blots of PP2A and eEF2K in MASMCs from aortas of *Nfatc3*$^{fl/fl}$ and *Nfatc3*^{smcKO} mice (n = 4). (E) Western blots of PP2A and eEF2K in MASMCs isolated from the aortas of *Nfatc3*-KI$^{fl/fl}$ and *Nfatc3*^{smcKI} mice (n = 4). (F) Mammalian two-hybrid analysis of the NFATc3 and eEF2 interaction. A pBIND plasmid encoding full-length NFATc3, the 1–415 aa, 416–

597 aa, and 598–1076 aa domains, and a pACT plasmid expressing full-length eEF2 were co-transfected into COS-7 cells. Luciferase activity was analyzed 48 h following transfection. (G) HEK293T cells were co-transfected with the His-eEF2 and Flag-NFATc3 plasmids for 48 h before a 24-h PDGF-BB treatment. Cell lysates were immunoprecipitated with an anti-Flag antibody, and His-eEF2 was determined using Western blot (n = 4). (H) Representative Western blots and quantification of ACTA2, CNN1, SM22α, and OPN in MASMCs transfected with vector or eEF2 plasmids and treated with TGF-β (10 μg/L) for 48 h (n = 4). (I) Western blots of puromycin incorporation assays and p-eEF2/eEF2 levels from MASMCs isolated from *Nfatc3*-KI$^{fl/fl}$ and *Nfatc3*^{smcKI} mice and treated with cabamiquine (2 μmol/L, 12 h) (n = 4). (J) Western blot and quantification of ACTA2, CNN1, SM22α, and OPN in MASMCs isolated from *Nfatc3*$^{fl/fl}$ and *Nfatc3*^{smcKO} mice and treated for 10 min with 10 μmol/L A484954 (n = 4). (K) Representative Western blots and quantification of ACTA2, CNN1, SM22α, and OPN in MASMCs isolated from the aortas of *Nfatc3*-KI$^{fl/fl}$ and *Nfatc3*^{smcKI} mice and treated with cabamiquine (2 μmol/L, 12 h) (n = 4). Data are presented as mean ± SD. (D–F) Unpaired Student's t-test was used. Two-tailed P-values are shown. (H–K) One-way ANOVA followed by Tukey's correction was used; adjusted P-values are shown. NS, no significance ($P > 0.05$).

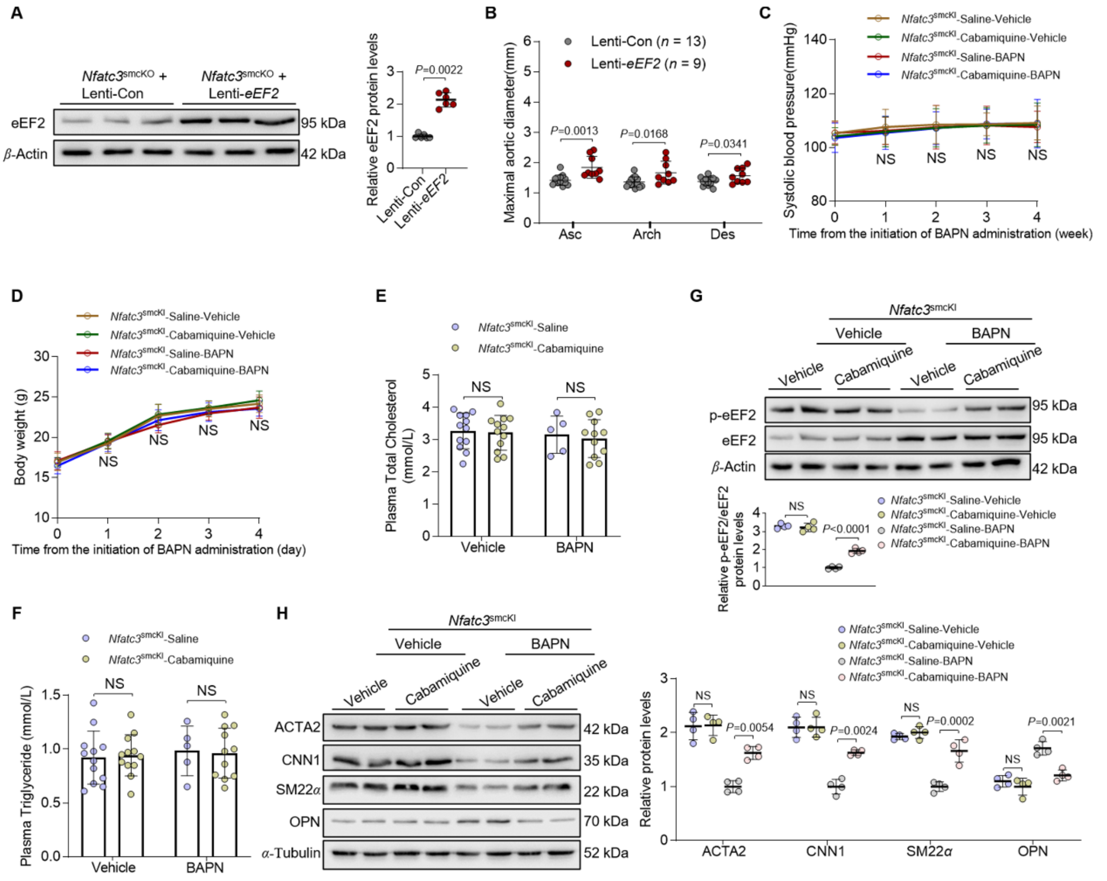

Figure S10 Enhanced contractile phenotype proteins of VSMCs in the aortas of *Nfatc3*^{smcKI} mice administered cabamiquine in the BAPN model. (A, B) Four-week-old male *Nfatc3*^{smcKO} mice were treated with control or eEF2-expressing lentivirus; 7 days after transfection, five-week-old mice were treated with BAPN (0.6 g/kg/day) for 28 days (n = 13). (A) Representative Western blots and quantification of eEF2 in MASMCs as indicated. (n = 6). (B) Maximum aortic diameter measurements. (C–H) Five-week-old male *Nfatc3*^{smcKI} mice were treated with vehicle (n = 12) or BAPN (n = 15) (0.6 g/kg/day) for 28 days. Saline and cabamiquine (3 mg/kg/day) were dosed orally for 28 days. (C–F) Systolic blood pressure (C), body weight (D), and TC (E) and TG (F) serum levels in mice as indicated (n = 12 for *Nfatc3*^{smcKI} mice treated with vehicle and saline or cabamiquine; n = 5 for *Nfatc3*^{smcKI} mice treated with BAPN and saline; n = 11 for *Nfatc3*^{smcKI} mice treated with BAPN and cabamiquine; mice that died of aortic rupture were not included in the measurements). (G) Western blots and quantification of p-eEF2 and eEF2 in aortic tissues treated as described above (n = 4). (H) Representative Western blots and quantification of ACTA2, CNN1, SM22α, and OPN in MASMCs as indicated (n = 4). Data are presented as mean ± SD. (A) Mann–Whitney U-test with the exact method; two-tailed P-values; (B) Unpaired Student's t-test was used. Two-tailed

P-values are shown. (C–H) Two-way ANOVA with Tukey's correction was used; adjusted *P*-values are shown. NS, no significance ($P > 0.05$).

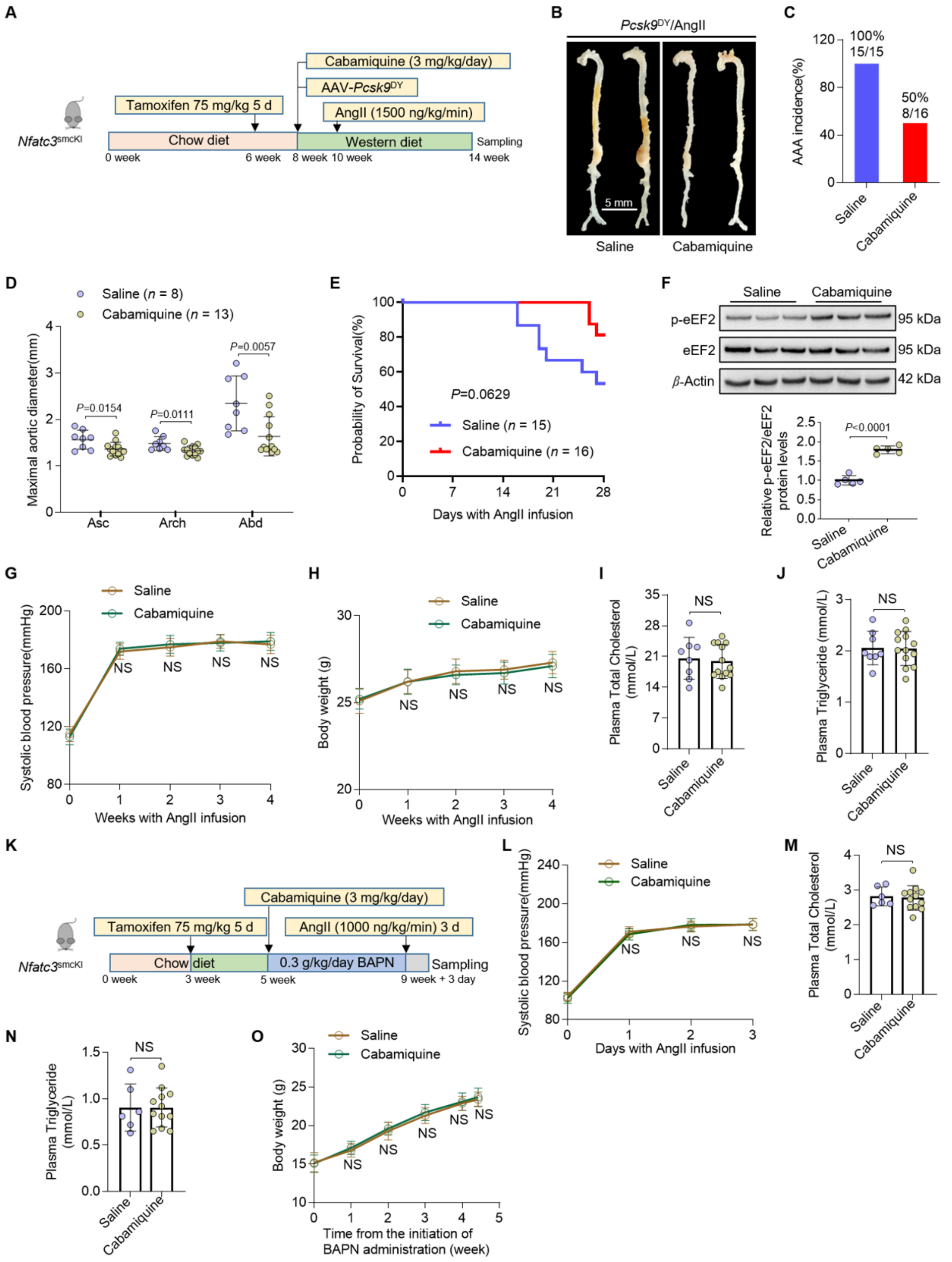

Figure S11 Cabamiquine mitigates the deleterious effects of NFATc3 overexpression on AAA development in the *Pcsk9*^{DY}/AngII model. (A–J) Eight-week-old *Nfatc3*^{smcKI} mice were injected intraperitoneally with AAV-*Pcsk9*^{DY} and fed a Western-type diet. After 2 weeks, they were infused with AngII (1500 ng/kg/min) for another 4 weeks.

Vehicle and cabamiquine (3 mg/kg/day) were dosed orally for 6 weeks (*n* = 15 for saline, *n* = 16 for cabamiquine). (A) Schematic of AAV-*Pcsk9*^{DY}/AngII model establishment. (B) Representative macrographs of the aorta. (C) AAA incidence. (D) Maximum aortic diameter measurements (*n* = 8 for saline, *n* = 13 for cabamiquine). (E) Survival rate. (F) Western blots and quantification of p-eEF2 and eEF2 in aortic tissues from *Nfatc3*^{smcKI} mice treated with AAV-*Pcsk9*^{DY}/AngII and saline (*n* = 5). (G–J) Systolic blood pressure (G), body weight (H), and TC (I) and TG (J) serum levels in mice as indicated (*n* = 8 for *Nfatc3*^{smcKI} mice treated with AAV-*Pcsk9*^{DY}/AngII and saline; *n* = 13 for *Nfatc3*^{smcKI} mice treated with AAV-*Pcsk9*^{DY}/AngII and cabamiquine; mice that died of aortic rupture were not included in the measurements). (K–O), Five-week-old *Nfatc3*^{smcKI} male mice were treated with BAPN (0.3 g/kg/day) for 28 days and infused with AngII (1000 ng/kg/min) for 3 days. Saline and cabamiquine (3 mg/kg/day) were dosed orally for 31 days (*n* = 17 for saline, *n* = 18 for cabamiquine). (K) Schematic of BAPN/AngII model establishment. (L–O) Systolic blood pressure (L), TC levels (M) and TG (N) serum levels, and body weight (O) in mice as indicated (*n* = 6 for *Nfatc3*^{smcKI} mice treated with BAPN/AngII and saline; *n* = 12 for *Nfatc3*^{smcKI} mice treated with BAPN/AngII and cabamiquine). Data are presented as mean ± SD. (D) Unpaired Student's *t*-test or Mann–Whitney U-test was used. (F–J, L–O) Unpaired Student's *t*-test was used. Two-tailed *P*-values are shown. (E) Log-rank tests were used. NS, no significance (*P* > 0.05).

Table S1 Characteristics of patients included in this study.

Characteristics	Control (*n* = 36)	TAAD (*n* = 36)	AAA (*n* = 8)
Age (years)	64.2 ± 8.4	62.3 ± 6.1	63.5 ± 4.1
Male (*n*, %)	21 (58.3)	31 (86.1)	7 (87.5)
Body mass index (kg/m^2)	23.82 ± 23	24.68 ± 2.1	25.03 ± 1.8
History of smoking (*n*, %)	14 (38.9)	8 (22.2)	3 (37.5)
Hypertension (*n*, %)	8 (22.2)	25 (69.4)	6 (75.0)
Diabetes mellitus (*n*, %)	4 (11.1)	5 (13.9)	1 (12.5)
Hyperlipidemia (*n*, %)	24 (66.7)	7 (19.4)	3 (37.5)
Maximum aortic diameter (cm)	NA	5.3 ± 1.6	6.3 ± 1.7

Data are presented as a number (percentage) or mean ± standard deviation. TAAD, thoracic aortic aneurysm and dissection; AAA, abdominal aortic aneurysm; NA, not available.

Table S2 Primers used in this study.

Gene	Forward primers (5′–3′)	Reverse primers (5′–3′)
Primers used for genotyping		
Nfatc3$^{fl/fl}$ F1	CATGGACAGCACATAATTGAGTGTGG	TCAACCGTAGACTTCATGATGCTCC
Nfatc3$^{fl/fl}$ F2	TTGGCTAACCTGGTCTGCTTGGT	CCAAGTCATCACTAGGCAATGGATCT
Nfatc3-KI$^{fl/fl}$ F1	GCATCTGACTTCTGGCTAATAAAG	GGGTGAGCATGTCTTTAATCTACC
Nfatc3-KI$^{fl/fl}$ F2	AAGCACGTTTCCGACTTGAGTTG	GGGTGAGCATGTCTTTAATCTACC
Myh11-cre	TGACCCCATCTCTTCACTCC	AGTCCCTCACATCCTCAGGTT
Primers used for RT-PCR		
NFATc3 (human)	GGTGGTCCCAAACCCTTTGAG	AAAAATTCCCGTTCTGGGTCAT
Nfatc3 (mouse)	GCTTTCTAGGTCGGAACGGAA	CGCCACAGTTTGCAGTAGTC
MMP2 (human)	TATTTGATGGCATCGCTCAG	GCCTCGTATACCGCATCAAT
Mmp2 (mouse)	GAACTTCCGATTATCCCATGATGAC	TCTGAGCGATGCCATCAAAGAC
MMP9 (human)	TGGGAAGTACTGGAGATTCT	CCTGTGTACACCCACACCTG
Mmp9 (mouse)	GCAGAGGCATACTTGTACCG	TGATGTTATGATGGTCCCACTTG
Mmp3 (mouse)	CATGAACTTGGCCACTCCCT	GTGGGTACCACGAGGACATC
Mmp13 (mouse)	TACCATCCTGCGACTCTTGC	TTCACCCACATCAGGCACTC
ACTB (human)	ATGGGTCAGAAGGATTCCTAT	AAGAGTGCCTCAGGGCAG
Actb (mouse)	GTTGGAGCAAACATCCCCCA	CGCGACCATCCTCCTCTTAG
Primers used for promoter construction		
*Mmp9*p (0 → −2.0 kb)	CTCGCTAGCCTCGAGGATATCACGGTGCTTGACACAGTAAAT	CAGTACCGGATTGCCAAGCTTGGTGGACTGGCGCTGTCT
*Mmp2*p (0 → −2.0 kb)	CTCGCTAGCCTCGAGGATATCCACCAAGCCCCACACTGGAA	CAGTACCGGATTGCCAAGCTTCTGCTGGGAAACATCTGTCGC
Primers used for plasmid construction		
His-*eEF2*	CTAGCGTTTAAACTTAAGCTTATGGTGAACTTCACAGTAGATCAGATC	AATGTTTCCACATGGCACGTCCTACAGTTTGTCCAGGAAGTTGTCC
eEF2 T56D	TCCTTGCGAGTGTCATCGAAGCGCGTCTCCCC	GGGGAGACGCGCTTCGATGACACTCGCAAGGA
eEF2 T56A	CTTGCGAGTGTCAGCGAAGCGCGTCTCCC	GGGAGACGCGCTTCGCTGACACTCGCAAG
pLV3-*eEF2*	CTGATACGAACTCGGAATTCGCCACCATGGTGAACTTCACAGTAGATCAG	TGGTCTTTGTAGTCGGATCCCAGTTTGTCCAGGAAGTTGTC
Flag-*Nfatc3*	GGTACCGAGGAGATCTGCCGCCGCGATCGCCATGACTACTGCAAACTGTGGC	AGTTTCTGCTCGAGCGGCCGCGTACGCGTCTGAGCACTGTGAGAGGTCATC
Flag-*Nfatc3* (1–415 aa)	CGACTCACTATAGGGCGGCCGGGAATTCGCCACCATGACTACTGCAAACTGTG	TCTTTATAATCACCGTCATGGTCTTTGTAGTCCTCGAGTGTGCGAAATATAGGGGTGTG
Flag-*Nfatc3* (416–597 aa)	CGACTCACTATAGGGCGGCCGGGAATTCGCCACCATGTCTTCATTACCTCCATTAGAC	TCTTTATAATCACCGTCATGGTCTTTGTAGTCCTCGAGAGCAGATCGCTGAGAGCACTC
Flag-*Nfatc3* (598–1076 aa)	CGACTCACTATAGGGCGGCCGGGAATTCGCCACCATGCAAGAACTCCCTCATATTGAG	CTTTATAATCACCGTCATGGTCTTTGTAGTCCTCGAGCTGAGCACTGTGAGAGGTCATC
Plasmid-*Nfatc3*-mutant (deletion of 416–597 aa)	CTACATATAAATGACCCAGAAAG<br>ATTTCGCACACAAGAACTCCCTCATATTGAG	GGAGTTCTTGTGTGCGAAATATAGGGGTGTG<br>GGATATCATTTGCTGCCAGATC

Primer sequences used for mammalian two-hybrid system		
pBIND-Flag-*NFATc3*	CGGGGATCCTTGCCACCATGACTACTGCAAACTGTGGCG	ATAGTTTAGCGGCCGCTCACTGAGCACTGTGAGAGGTC
pBIND-Flag-*NFATc3* (1-415 aa)	CGGGGATCCTTGCCACCATGACTACTGCAAACTGTGGCG	ATAGTTTAGCGGCCGCTCATGTGCGAAATATAGGGGTGTGGCC
pBIND-Flag-*NFATc3* (416-597 aa)	CGGGGATCCTTGCCACCATGTCTTCATTACCTCCATTAGACTGGCC	ATAGTTTAGCGGCCGCTCAAGCAGATCGCTGAGAGCACT
pBIND-Flag-*NFATc3* (598-1076 aa)	CGGGGATCCTTGCCACCATGCAAGAACTCCCTCATATTGAGAAGTACAGT	ATAGTTTAGCGGCCGCTCACTGAGCACTGTGAGAGGTC
pACT-*eEF2*	CCGGAATTCGCCACCATGGTGAACTTCACAGTAGATCAGATCCG	ATAGTTTAGCGGCCGCCTACAGTTTGTCCAGGAAGTTGTCCA
Primers used for ChIP-qPCR		
*Mmp2*p-*Nfatc3* (Site: ATGGAAACT)	AGGGAGCAGTCACTACTCAACTT	TCCCAAAAATCTGGCAATACTGGATGC
*Mmp9*p-*Nfatc3* (Site: GTGGAATTC)	TGAAGATTCAGCCTGCGGAAGAC	CTGACAGCCTTCTTTGACTCAGCT

Table S3 Study design for animal experiments.

Mouse group	Sex	Age (week)	Number (prior to experiment)	Number (after termination)	Littermates (Yes/No)
wild-type +Saline	Male	5	8	8	No
wild-type +BAPN/AngII	Male	5	8	5	No
wild-type +*Pcsk9*^{DY}/AngII	Male	8	8	7	No
NFATc3 wild-type (*Nfatc3*$^{fl/fl}$) +Saline	Male	5	12	12	No
NFATc3 VSMC-knockout (*Nfatc3*^{smcKO}) +saline	Male	5	12	12	No
NFATc3 wild-type (*Nfatc3*$^{fl/fl}$) + BAPN/AngII	Male	5	22	15	No
NFATc3 VSMC-knockout (*Nfatc3*^{smcKO}) + BAPN/AngII	Male	5	22	22	No
NFATc3 Wild-type (*Nfatc3*$^{fl/fl}$) + *Pcsk9*^{DY}/AngII	Male	8	20	17	No
NFATc3 VSMC-knockout (*Nfatc3*^{smcKO}) + *Pcsk9*^{DY}/AngII	Male	8	21	21	No
Rosa26-NFATc3 (*Nfatc3*-KI$^{fl/fl}$) +saline	Male	5	14	14	No
NFATc3 VSMC-knockin (*Nfatc3*^{smcKI}) +saline	Male	5	14	14	No
Rosa26-NFATc3 (*Nfatc3*-KI$^{fl/fl}$) + BAPN/AngII	Male	5	19	13	No
NFATc3 VSMC-knockin (*Nfatc3*^{smcKI}) + BAPN/AngII	Male	5	20	7	No
Rosa26-NFATc3 (*Nfatc3*-KI$^{fl/fl}$) + *Pcsk9*^{DY}/AngII	Male	8	19	17	No
NFATc3 VSMC-knockin (*Nfatc3*^{smcKI}) + *Pcsk9*^{DY}/AngII	Male	8	19	10	No
NFATc3 Wild-type (*Nfatc3*$^{fl/fl}$) +BAPN	Male	5	15	10	No
NFATc3 VSMC-knockout (*Nfatc3*^{smcKO}) + BAPN	Male	5	15	15	No
Rosa26-NFATc3 (*Nfatc3*-KI$^{fl/fl}$) + BAPN	Male	5	17	12	No
NFATc3 VSMC-	Male	5	18	6	No

knockin (*Nfatc3*^{smcKI}) + BAPN					
NFATc3 VSMC-knockout (*Nfatc3*^{smcKO}) + vehicle + Lenti-Con	Male	5	13	13	No
NFATc3 VSMC-knockout (*Nfatc3*^{smcKO}) + vehicle + Lenti-eEF2	Male	5	13	13	No
NFATc3 VSMC-knockout (*Nfatc3*^{smcKO}) + BAPN+ Lenti-Con	Male	5	13	13	No
NFATc3 VSMC-knockout (*Nfatc3*^{smcKO}) + BAPN + Lenti-eEF2	Male	5	13	9	No
NFATc3 VSMC-knockin (*Nfatc3*^{smcKI}) + BAPN+ saline	Male	5	15	5	No
NFATc3 VSMC-knockin (*Nfatc3*^{smcKI}) + BAPN + cabamiquine	Male	5	15	11	No
NFATc3 VSMC-knockin (*Nfatc3*^{smcKI}) + BAPN/AngII + Saline	Male	5	17	6	No
NFATc3 VSMC-knockin (*Nfatc3*^{smcKI}) + BAPN/AngII + cabamiquine	Male	5	18	12	No
NFATc3 VSMC-knockin (*Nfatc3*^{smcKI}) + *Pcsk9*^{DY}/AngII + saline	Male	8	15	8	No
NFATc3 VSMC-knockin (*Nfatc3*^{smcKI}) + *Pcsk9*^{DY}/AngII +cabamiquine	Male	8	16	13	No
